# Topological-Projection-Consistent Keypoint Extraction for PnP-Based Space-Target Pose Measurement Under Extreme Illumination

**DOI:** 10.3390/s26196079

**Published:** 2026-09-25

**Authors:** Jiuzheng Song, Zhen Liu, Yiming Su, Chonglin Zhao

**Affiliations:** Key Laboratory of Precision Opto-Mechatronics Technology, Ministry of Education, Beihang University, Haidian, Beijing 100191, China; songjiuzheng@buaa.edu.cn (J.S.); ymsu23@buaa.edu.cn (Y.S.); by2317320@buaa.edu.cn (C.Z.)

**Keywords:** space-target pose measurement, topological projection consistency, keypoint detection, extreme illumination, Perspective-n-Point

## Abstract

Accurate Perspective-n-Point (PnP) pose measurement for space targets benefits from consistency between the topological structure of extracted two-dimensional feature points and that of projected three-dimensional template points. Existing keypoint extraction methods mainly optimize individual point localization accuracy, while paying insufficient attention to the global topological consistency of the keypoint set under relative-position relations, structural dependencies, and rigid projection constraints. As a result, frontend keypoint errors can be further amplified by the backend PnP solver. To address this problem, this paper proposes a topological-projection-consistent keypoint extraction method for PnP-based space-target pose measurement under extreme illumination. First, a Residual-Gated Context-Chained Enhancement Module (RG-CCEM) is introduced to build global structural feature propagation in degraded images. During distillation, both two-dimensional pairwise structural constraints and three-dimensional weak-perspective template reprojection constraints are imposed so that the student network learns keypoint sets whose projected topology remains consistent with the rigid 3D template. A reliability–reprojection-residual jointly reweighted PnP method is then used to reduce the influence of topologically inconsistent correspondences on pose estimation. Experiments show that the proposed method improves the stability of keypoint topology and pose measurement accuracy under extreme illumination. Compared with the DETRPose baseline, the proposed method decreases the outlier-keypoint ratio from 21.0% to 9.2%, and reduces rotation, translation, and camera-center RMSE by approximately 55.1%, 39.8%, and 56.3%, respectively, on our laboratory semi-physical dataset. Limitations of this method are further discussed.

## 1. Introduction

Space-target pose measurement is important for autonomous rendezvous and docking, on-orbit servicing, and related space missions. Monocular vision is attractive because it offers flexible deployment with relatively low payload and power requirements; in a Perspective-n-Point (PnP) pipeline, the pose is recovered from correspondences between two-dimensional (2D) image keypoints and a three-dimensional (3D) target template [1,2,3,4,5]. For a rigid target, accurate localization of isolated keypoints is not sufficient: the complete keypoint set should also preserve a relative topology that can be jointly explained by the projection of one fixed 3D template under a common camera model [6]. Recent work on spacecraft pose estimation with feature degradation shows that degraded observations can produce missing, blurred, or spatially inconsistent geometric cues [7]. In practical observations, glare, blooming, lens flare, overexposure, and specular reflection may further obscure structural features [8]. These effects make it difficult to maintain consistency between extracted 2D keypoints and projected 3D template points under extreme illumination.

To improve robustness under degraded observations, existing studies have explored multi-sensor or stereo sensing, image enhancement, geometric supervision, and domain adaptation [7,9,10,11,12]. These directions can improve sensing diversity or compensate for appearance degradation, but they may also introduce additional hardware, calibration requirements, or training assumptions. A detailed discussion of these research directions is provided in Section 2. In this paper, we focus on a monocular point-feature pipeline because the detected keypoints can be directly associated with a known 3D template for subsequent PnP solving, and the main challenge is to preserve their global projection geometry under extreme illumination.

Traditional point-feature extraction methods are sensitive to illumination variations and show limited robustness under complex lighting. In contrast, deep-learning-based keypoint detectors provide stronger feature representation and generalization, and can partially adapt to image variations caused by complex illumination [3,13]. However, most learning-based keypoint detection methods take the localization accuracy of each individual keypoint as the primary optimization objective. Their network designs and loss functions are usually centered on pixel-level coordinate errors or heatmap response errors, while explicit constraints on the global topology of the target keypoint set are insufficient. Some methods introduce long-range dependencies among keypoints through Transformer blocks, self-attention, or global context modeling, but these global features mainly serve semantic representation or detection robustness and do not directly guarantee topological projection consistency between the extracted keypoint set and the projected 3D template points. Moreover, these methods usually assume that structural features in the image are clearly visible. Under extreme illumination, missing local features, false responses, and keypoint shifts can make the relative topology of the extracted keypoints deviate from the true projection structure. In this case, even if several local keypoint predictions appear reasonable, the whole keypoint set may still be impossible to explain consistently with a single rigid projection model, which in turn reduces the accuracy of PnP-based pose measurement or even causes pose-solving failure.

In addition, keypoint detection errors under extreme illumination are usually heteroscedastic [14,15]. Different keypoints are affected to different degrees by overexposure, strong reflection, shadow occlusion, and local texture loss, and therefore differ in observation reliability and error direction. Such errors do not act uniformly on the entire keypoint set. Instead, they may break the relative topological relations among keypoints and reduce the topological projection consistency between the extracted point set and the projected 3D template. If all 2D–3D correspondences are still assigned equal weights in PnP reprojection optimization, keypoints with low reliability or large topological shifts may have excessive influence on the solution, causing pose estimation to converge to an incorrect projection relation. From the perspective of the Gauss–Markov theorem, a more reasonable strategy is to weight different keypoints according to their observation variance and reprojection residuals, so that reliable keypoints with stable topological relations play a dominant role in pose-solving.

Under extreme illumination, the central challenge is not merely to localize each keypoint independently but to recover a set of 2D correspondences that can be jointly explained by a single pose of a fixed rigid 3D template. We therefore formulate space-target pose measurement as a geometry-preserving correspondence problem and propose a unified topological-projection-consistent framework. In this work, topological-projection consistency denotes the simultaneous preservation of relative keypoint relationships and the compatibility between the extracted 2D keypoint set and the projection of a fixed 3D template under a common camera model. Unlike conventional pipelines that optimize keypoint localization and PnP estimation as largely independent stages, the proposed framework carries this geometric constraint from data supervision to feature learning, and finally to pose optimization.

At the supervision level, a projection-consistent data augmentation strategy generates paired clear-domain and degraded-domain samples while preserving the shared camera pose, 3D template, 2D keypoints, masks, and visibility relationships. At the feature-learning level, a cross-illumination-domain teacher–student network transfers reliable structural knowledge from clear images to degraded images. The network combines illumination adaptation and RG-CCEM-based global context propagation with pairwise 2D structural distillation and 3D-template weak-perspective reprojection regularization, thereby encouraging the student to predict keypoint sets that remain compatible with the rigid target structure. The weak-perspective model is used only as a stable training regularizer, whereas the final metric pose is recovered using the full-perspective PnP model. At the pose-optimization level, a reliability–reprojection-residual jointly reweighted PnP method integrates learned point-wise observation reliability with geometry-based reprojection residuals, reducing the influence of unreliable or geometrically inconsistent correspondences.

The main contributions of this paper are as follows:

(1) A projection-consistent cross-illumination data supervision strategy is proposed. Unlike conventional image-level augmentation, the proposed strategy preserves the synchronized projection relationships among the image, target template, keypoints, masks, camera parameters, and visibility cues when generating degraded samples. It therefore provides illumination-diverse training data without corrupting the underlying 2D–3D correspondence geometry.
(2) A topology-aware cross-illumination teacher–student keypoint network is developed. The network combines a Light Adapter and RG-CCEM to propagate reliable global structural information from visible regions to locally degraded regions. Its distillation objective jointly imposes pairwise 2D structural constraints and weak-perspective reprojection constraints derived from the fixed 3D template, explicitly encouraging globally compatible keypoint configurations rather than optimizing isolated point locations only.
(3) A reliability–reprojection-residual jointly reweighted full-perspective PnP method is introduced. By combining the student-predicted keypoint reliability with the reprojection residual of each correspondence, the proposed backend links image-domain observation quality with pose-domain geometric consistency and suppresses the influence of unreliable or topologically shifted keypoints during pose estimation.

Together, these components establish a unified geometry-consistency principle for robust keypoint extraction and PnP-based pose measurement under extreme illumination.

## 2. Related Work

To address keypoint-topology degradation and backend pose-error amplification in space-target pose measurement under extreme illumination, existing studies can be broadly grouped into three categories: directly training keypoint models on degraded images or mixed-domain data, enhancing or restoring images before training or measurement, and transferring structural information from clear-domain images or stronger models to the degraded domain through knowledge distillation. These methods alleviate degradation from the perspectives of network learning, input restoration, and knowledge transfer, respectively. However, they still have clear limitations in degraded-domain annotation reliability, topological projection consistency, and keypoint-uncertainty modeling.

### 2.1. Direct Keypoint Training Methods

Direct training methods usually adopt either a “keypoint detection + PnP” framework or an end-to-end pose regression framework to estimate the relative pose of a space target from monocular images. Xu et al. used a deep keypoint detection network for space-target pose measurement, demonstrating the explicit geometric interpretability of keypoint-based methods [3]. Ye et al. introduced a dual-channel Transformer to enhance global feature representation for non-cooperative target pose estimation [13]. The SPEED and SPEED+ datasets further promoted this field and revealed the significant domain gap among synthetic images, laboratory images, and real space images [16,17]. Park and D’Amico addressed cross-domain problems from the perspectives of multi-task learning and online refinement [12]. These methods can perform well when visible features are sufficient and annotations are reliable, but their performance remains highly dependent on the training-data distribution and keypoint annotation quality. Representative keypoint detectors, such as RTMPose and DETRPose, also commonly assume that image features are stable and visible, which is not always valid under extreme illumination [18,19].

In addition to direct training, some methods expand training samples through data augmentation. However, conventional image-level augmentations, such as mosaic combination, geometric transformation, and brightness perturbation, cannot eliminate the influence of local degradation on keypoint observations, which directly affects topological projection consistency [20,21]. Some image-reconstruction- or rendering-based data generation methods are constrained by the collected backgrounds and therefore have reduced data generalization [22]. Neural Radiance Fields (NeRFs) and 3D Gaussian Splatting (3DGS) can replace backgrounds and render targets in more scenes, but if the rendering process does not preserve the perspective projection relation, additional errors may be introduced into pose measurement [23,24,25].

Under extreme illumination, the core limitation of direct training methods lies in the inconsistency between supervision signals and real geometric observations. Local overexposure, strong reflection, and shadow occlusion can weaken or even remove visible structures around keypoints, making manual annotations or pseudo-labels inevitably subjective and biased. During training, the network may learn not only the target structure but also annotation noise. Existing studies on feature degradation also show that structural-information loss in degraded images directly affects space-target pose estimation [18,19,26].

At the network level, traditional convolutional structures mainly focus on local texture mining. They have limited adaptability to texture-missing regions caused by strong light and shadows, and do not explicitly consider the topological structure among keypoints [26]. Transformers model long-range dependencies through self-attention and thus provide a stronger global modeling capability [27]. The recently proposed Mamba [28] and VMamba [29] further achieve global dependency modeling with linear complexity. Nevertheless, the learning objectives of these networks are usually still built on 2D image features and lack explicit rigid geometric constraints. For keypoint detection, a network must preserve local details such as corners and edges while maintaining the global topological relation of the rigid target structure [15,24,26,27].

Moreover, different keypoints are affected by degradation to different degrees. If a uniform loss is used for all keypoints, it is difficult to distinguish high-reliability observations from low-reliability observations or to explicitly model observation differences among keypoints. The backend PnP solver further amplifies this problem. EPnP and SQPnP provide efficient foundations for estimating pose from 2D–3D correspondences [4,5]. EPro-PnP, uncertainty-aware PnP, and conformal keypoint detection further show that keypoint errors and their uncertainty directly affect the stability of pose solutions [14,15,30]. However, many direct training methods still feed network-predicted keypoints into PnP with equal weights, without sufficiently considering differences in degradation degree, observation variance, and geometric contribution among keypoints. Consequently, under severe image degradation, keypoint errors and pose errors are often amplified simultaneously.

### 2.2. Image Enhancement Preprocessing Methods

This category of methods first enhances or restores degraded images, and then performs keypoint detection and pose-solving. Representative works in low-light enhancement and exposure correction include Zero-DCE, which performs low-light enhancement through zero-reference curve estimation [31]; Restormer, which uses a Transformer architecture to improve high-resolution image restoration [32]; Retinexformer, which combines Retinex priors and Transformer modeling for complex illumination [33]; region-aware exposure consistency correction, which handles mixed-exposure images containing both local overexposure and underexposure [10]; and Color Shift Estimation-and-Correction (CSEC), which estimates and corrects color and exposure shifts and can be used as exposure-correction preprocessing before keypoint detection [11]. These methods can improve visual quality and enhance the visibility of some textures and edges.

However, for space-target pose measurement, image enhancement quality is not equivalent to geometric measurement accuracy. Metallic thermal-control coatings and solar panels on space targets can easily produce strong reflections and saturated overexposure. Once the true edge information in saturated regions is lost during imaging, a 2D enhancement network can only infer the missing structures from image priors. In this case, the enhanced image may appear clearer, but the true projected keypoint positions are not guaranteed. If these artificially inferred textures or edges are treated as valid features by a downstream keypoint network, the errors will continue to propagate into PnP reprojection optimization.

### 2.3. Knowledge Distillation and Structural Constraint Methods

Knowledge distillation transfers soft labels, feature responses, or intermediate structural knowledge from a teacher network to a student network for performance improvement or model compression. The classical distillation formulation was first used for model compression and generalization enhancement [34], and subsequent work developed more fine-grained forms of distillation losses [35]. In pose estimation tasks, distillation usually requires stronger structural constraints than classification. For example, online pose distillation learns implicit keypoint relations through multi-branch heatmaps [36], while 6D pose distillation improves local geometric representation by aligning local prediction distributions [37]. These studies indicate that distillation for keypoint localization and pose estimation cannot merely transfer class-level or global semantic information; it must also consider local structures and spatial relations.

In degraded scenarios, distillation has the advantage of using clear images or stronger teacher networks to provide more stable structural knowledge, but its limitations are also evident. First, generic distillation methods are mostly designed for classification, detection, or general pose estimation tasks, and usually do not include 3D keypoint topology constraints for space targets. Second, keypoint visibility varies significantly in degraded images. If the same distillation strength is applied to all keypoints or feature regions, uncertain teacher responses in invisible regions may also be transferred to the student. Third, if the frontend confidence obtained through distillation is not linked to backend geometric solving, the mismatch between keypoint reliability and PnP weighting remains difficult to resolve.

In summary, existing methods improve visual measurement under degraded conditions from the perspectives of direct training, image enhancement, and knowledge transfer. Nevertheless, three common problems remain: annotations and pseudo-labels for degraded images are insufficiently reliable; image enhancement or generation results lack consistency constraints at the keypoint-projection level; and frontend keypoint uncertainty and backend PnP pose optimization are not modeled in a unified manner. These problems are major causes of reduced accuracy and robustness in space-target pose measurement under severe image degradation.

## 3. Method

This section establishes the space-target keypoint detection and pose-solving pipeline around feature-level topological projection consistency. The basic idea is as follows. First, projection-consistent augmentation is used to construct clear-domain and degraded-domain samples that share the same 3D template projection relation. Then, a teacher–student network transfers stable keypoint structural relations from clear images to extreme-illumination images. Finally, the predicted keypoints and their reliability values are fed into a reliability–reprojection-residual jointly reweighted PnP solver for pose estimation. The proposed pipeline does not simply improve image visibility or individual point localization accuracy. Instead, it jointly preserves the topological consistency between the 2D keypoint set and the projected 3D template point set at the data, network, and backend-optimization levels. The overall framework is shown in Figure 1.

Let the 3D keypoint template of the space target be $X={\left\{X_{i}\in R^{3}\right\}}_{i=1}^{N}$. Given camera intrinsics K, distortion parameters d, and target pose (R,t), the projection of the *i*-th 3D template point on the image plane is

(1) $$u_{i}=\Pi \left(X_{i},K,d,R,t\right),$$

where Π(·) denotes the projection function including lens distortion. In this paper, topological projection consistency means that the 2D keypoint set predicted by the network should be explainable as the projection of the same 3D template X under a single camera pose, rather than as several mutually independent local 2D responses.

### 3.1. Projection-Consistent Data Augmentation

Extreme illumination can destroy true textures and edges around some semantic keypoints through overexposure, shadows, or strong reflection, as shown in Figure 2. In this situation, directly annotating degraded images manually introduces obvious subjective errors. If these errors are used for training, the network may learn local false responses in degraded images rather than the true projection structure of the space target. To reduce dependence on unreliable degraded-domain labels, this paper first recovers camera poses and stable 3D keypoints from mildly degraded images, and establishes a traceable 3D template through Structure from Motion (SfM)/reconstruction results [38]. The foreground target and background are then rendered separately in a unified geometric coordinate system to generate clear-domain samples, while degraded-domain samples are generated under the same camera pose and 3D template constraints. The resulting training samples not only increase the number of images, but more importantly preserve a shared topological projection relation among clear images, degraded images, masks, and keypoint labels.

#### 3.1.1. Mild-Degradation Sample Selection

As shown in Figure 1, mildly degraded samples are first selected after data acquisition. The selection criterion is not whether the image is completely ideal but whether the keypoint topology remains distinguishable, the target contour is complete, the overall imaging is stable, and the image can be successfully reconstructed by COLMAP/SfM. Samples whose reconstruction reprojection errors are below a predefined threshold are regarded as reliable geometric anchors for subsequent data augmentation. The purpose is to establish the 3D template and projection relation from a small number of trustworthy samples, rather than learning unstable annotations directly from severely degraded images.

#### 3.1.2. Clear-Domain Sample Augmentation

For each mildly degraded sample, the target foreground is first obtained through segmentation. The reconstructed image sequence and 3DGS are then used to obtain the target representation $G_{\mathrm{obj}}$ and background representation $G_{\mathrm{bg}}$, respectively, as shown in Figure 1(2). A differentiable rendering operator R is used to generate clear reference images from novel viewpoints:

(2) $$I_{\mathrm{clear}}=R\left(G_{\mathrm{obj}},G_{\mathrm{bg}};\Theta_{\mathrm{view}}\right),$$

where $\Theta_{\mathrm{view}}$ contains the view pose, camera intrinsics, and distortion parameters. Since the image, visible-region mask, and 2D keypoint labels are generated synchronously from the same view parameters, all clear-domain samples naturally satisfy the following geometric matching constraint:

(3) $$\left(u_{i}^{\mathrm{clear}},M^{\mathrm{clear}}\right)=\left(\Pi \left(X_{i},K,d,R,t\right),V\left(G_{\mathrm{obj}};\Theta_{\mathrm{view}}\right)\right),$$

where $M^{\mathrm{clear}}$ denotes the visible target-region mask and V(·) denotes the mask rendering function. Although the appearance of the clear image changes with the viewpoint, the keypoint and mask labels are always determined by the same geometric truth. Therefore, the purpose of this augmentation is not merely to expand the texture distribution but to construct a structural supervision source without projection mismatch.

#### 3.1.3. Degraded-Domain Sample Rendering

Extreme-illumination degradation is not simply treated as random noise in this paper. Instead, irradiation, reflection, halo, and scattering effects are modeled according to the proxy geometry of the space target. The proxy geometry P contains keypoint topology, surface adjacency, and the visible range of each target region, and is used to approximate strong-illumination degradation in space-target imaging. The degraded sample $I_{\deg}$ is defined as

(4) $$I_{\deg}=D\left(I_{\mathrm{clear}},P,\Theta_{\mathrm{light}}\right),$$

where D is the degradation rendering operator, and $\Theta_{\mathrm{light}}$ contains degradation-control parameters such as illumination intensity, incident direction, reflection gain, halo scale, and local scattering coefficient. The proxy geometry and degraded rendering result are shown in Figure 3. Unlike random 2D augmentation, clear and degraded samples share camera parameters and a 3D keypoint template. Therefore, the corresponding keypoints satisfy

(5) $$u_{\mathrm{clear},i}=u_{\deg,i}=\Pi \left(X_{i},K,d,R,t\right).$$

The proxy geometry P is a semantic lighting scaffold constructed once for each target from the reconstructed 3D keypoints. The construction comprises four stages. Stage 1: Keypoint anchoring:Multi-view images are registered by COLMAP/SfM, and the annotated semantic keypoints are triangulated in a common coordinate frame. Stage 2: Topology definition: Reflective body and panel patches are connected according to the fixed keypoint topology. Stage 3: Surface completion: Each patch is subdivided and interpolated, with small normal displacements applied to non-keypoint vertices while the anchored keypoints remain fixed. Stage 4: Rendering-cue derivation: Surface normals, viewing directions, and depth ordering are computed for the degradation operator D.

The supervision labels are generated independently as $\Pi (X_{i},K,d,R,t)$. Proxy inaccuracies therefore do not alter the 2D labels or the 3D template used by PnP; they affect only the rendered appearance, including highlights, saturation, depth ordering, and approximate occlusion boundaries.

The final paired sample is denoted as $S=\{I_{\mathrm{clear}},I_{\deg},X,M,\Theta_{\mathrm{view}}\}$. For real strong-light images without accurate labels, adjacent mildly degraded frames are used as geometric anchors and assigned smaller loss weights, which preserves real degradation appearance while reducing annotation-noise propagation. In this way, the supervision received by the student network is not a set of isolated 2D points but a keypoint projection topology consistent with the 3D template and view parameters.

### 3.2. Teacher–Student Keypoint Network for Topological Projection Consistency

This paper adopts a teacher–student framework based on DETRPose [19]. The teacher network is trained on clear-domain samples and then frozen. It provides class responses, decoder keypoints, query features, and keypoint structural relations. The student network takes extreme-illumination degraded images as input and introduces a Light Adapter and a Residual-Gated Context-Chained Enhancement Module (RG-CCEM) to perceive local observation quality and propagate global structural information. During inference, only the student network is deployed. In addition to keypoints, the student predicts point-wise keypoint reliability $r_{i}^{\mathrm{kpt}}$ for backend PnP weighted optimization. The complete teacher-student framework for topological projection consistency is shown in Figure 4.

The core objective of the network is not to simply restore local textures but to preserve the projection topology of the keypoint set when local features are missing. Extreme illumination may produce strong reflection, saturation, or shadows in local regions, and local responses may become sharp but geometrically incorrect. If the detector treats all local responses equivalently, it may generate 2D–3D correspondences that appear semantically reasonable but do not satisfy the projection relation of the rigid template as a whole. Therefore, the student network is designed to estimate local illumination/degradation reliability, propagate global target structural information from reliable regions to degraded regions through RG-CCEM, and constrain the keypoint set to be consistent with the projected 3D template topology through structural distillation.

#### 3.2.1. Light Adapter

The Light Adapter is shown in Figure 5a. Its role is to provide a local observation-quality prior for subsequent topological-structure learning. This module does not directly aim to restore a degraded image to a clear image. Instead, it estimates an illumination confidence map $C_{\mathrm{illum}}$ from brightness, chromaticity, local texture variance, and large-area degradation statistics. Let the image brightness be L, the chromaticity difference be C, and the local brightness variance be $V_{w}\left(L\right)$. The soft responses for overexposed, underexposed, and flat low-texture regions are

(6) $$\begin{array}{cc} p_{o} & =\sigma \left((L-\tau_{o})/T\right)\sigma \left((\tau_{s}-C)/T\right),\\ p_{u} & =\sigma \left((\tau_{u}-L)/T\right),\\ p_{f} & =\sigma \left(\left(\tau_{v}-V_{w}\left(L\right)\right)/T_{v}\right)\sigma \left(\left(\tau_{c}-C\right)/T_{c}\right), \end{array}$$

where σ(·) is the sigmoid function, $\tau_{\cdot }$ denotes degradation thresholds, and $T_{\cdot }$ denotes softening temperatures. The degradation map is defined as $D_{\mathrm{illum}}=\max\left(p_{o},p_{u},p_{f},p_{r}\right)$, where $p_{r}$ denotes the large-area degradation response obtained by window averaging. The illumination confidence map is $C_{\mathrm{illum}}=1-D_{\mathrm{illum}}$. At the input side, a zero-initialized residual branch performs lightweight compensation, as follows:

(7) $$I_{\mathrm{ad}}=I_{\deg}+A_{\mathrm{in}}\left(I_{\deg}\right),$$

where $A_{\mathrm{in}}\left(\cdot \right)$ consists of convolution, depthwise convolution, and channel attention. In the backbone and encoder, illumination-aware feature modulation is further introduced:

(8) $$F_{\mathrm{conf}}^{\left(l\right)}=\Phi^{\left(l\right)}\left(F^{\left(l\right)};C_{\mathrm{illum}},c,h\right),$$

where *c* is the coarse illumination category and h is the global illumination context vector. This design avoids treating extreme illumination as an equivalent pixel-restoration problem. Instead, it modulates feature extraction according to local observation quality, enabling the network to distinguish reliable and unreliable regions during subsequent structural propagation.

From the perspective of topological projection consistency, the Light Adapter prevents erroneous local responses in degraded regions from excessively participating in structural inference. In overexposed or shadowed regions, increasing visual contrast does not necessarily recover the true contour position and may even introduce artificial edges. Therefore, the Light Adapter only provides an observation-quality prior and mild feature compensation, making the subsequent RG-CCEM more likely to propagate structural information from reliable regions rather than blindly strengthening pseudo-features in degraded regions.

#### 3.2.2. Residual-Gated Context-Chained Enhancement Module

RG-CCEM is the feature-bearing module for maintaining topological projection consistency. It extracts global structural cues from illumination-modulated multi-scale features. First, channel compression suppresses redundant degraded textures. Then, content-driven prompts and global attention enhance target contours and corner-neighborhood features. Finally, gated residual addition restores the channel dimension. For the *l*-th feature level, the module is formulated as

(9) $$\begin{array}{cc} Z^{\left(l\right)} & =\phi_{\mathrm{red}}\left(F_{\mathrm{conf}}^{\left(l\right)}\right),\\ \Delta F^{\left(l\right)} & =\phi_{\mathrm{up}}\left(\mathrm{CCEM}\left(Z^{\left(l\right)},P^{\left(l\right)}\right)\right),\\ F_{\mathrm{enh}}^{\left(l\right)} & =F_{\mathrm{conf}}^{\left(l\right)}+\gamma^{\left(l\right)}\Delta F^{\left(l\right)}, \end{array}$$

where $\phi_{\mathrm{red}}$ and $\phi_{\mathrm{up}}$ denote channel compression and channel restoration, respectively, $P^{\left(l\right)}$ is the learnable prompt, and $\gamma^{\left(l\right)}$ is a zero-initialized gate coefficient. The enhanced features are fed into the encoder and decoder to predict class, keypoints, and reliability.

RG-CCEM is introduced because keypoints on a space target are not independent local points but the projected structure of the same rigid template on the image plane. When a corner or solar-panel edge is corrupted by strong light, its location can still be jointly constrained by the visible body contour, the opposite solar-panel edge, and the global target layout. RG-CCEM propagates structural cues from reliable regions to degraded regions through global attention, enabling the network to compensate for missing local features according to the overall target topology. The gated residual design prevents the enhanced branch from overwriting useful original features, so the module behaves as an adaptive structural compensation term. For the backend PnP solver, a small number of topologically inconsistent keypoints can cause large pose deviations. Therefore, global structural propagation is critical for maintaining topological projection consistency at the feature level.

RG-CCEM contains three architectural choices designed to propagate reliable structural information under degraded imaging conditions. First, channel projection maps $F_{\mathrm{conf}}^{\left(l\right)}$ to a compact subspace $Z^{\left(l\right)}=\phi_{\mathrm{red}}\left(F_{\mathrm{conf}}^{\left(l\right)}\right)$ before global context modelling. This reduces redundant responses from overexposed, shadowed, or reflection-dominated regions and prevents them from overwhelming the subsequent context-enhancement operation. Second, a learnable prompt $P^{\left(l\right)}$ is incorporated into the context-chained enhancement module, allowing the enhancement branch to introduce target-structure cues in addition to image-derived features when local evidence is unreliable. Third, the enhanced feature is injected through a zero-initialized residual gate $\gamma^{\left(l\right)}$, so the module starts as an identity mapping and gradually introduces only the residual information supported by training. Together with the illumination-confidence features produced by the Light Adapter, these choices enable global structural compensation while preserving the original local representation. Thus, unlike purely local enhancement or direct feature recalibration, RG-CCEM propagates information from reliable regions to degraded regions without simply amplifying the strongest local responses.

#### 3.2.3. Topological-Projection-Consistent Distillation Losses

The distillation losses jointly align class responses, keypoint coordinates, decoder queries, intermediate features, reliability, and target structure, as shown in Figure 4. The core terms that directly express topological projection consistency are pairwise structural distillation and 3D-template weak-perspective reprojection constraints. Let ${\hat{u}}_{i}^{t}$ be the *i*-th keypoint predicted by the teacher network on the clear image, and let $u_{i}^{s}$ be the *i*-th keypoint predicted by the student network on the degraded image. To preserve rigid topology under degradation, pairwise structural distillation is first introduced:

(10) $$L_{\mathrm{pair}}=\frac{\sum_{i,j}w_{ij}{∥d_{ij}^{s}-d_{ij}^{t}∥}_{1}}{\sum_{i,j}w_{ij}+\epsilon },$$

where $d_{ij}^{s}=u_{i}^{s}-u_{j}^{s}$ and $d_{ij}^{t}={\hat{u}}_{i}^{t}-{\hat{u}}_{j}^{t}$. This loss does not constrain an isolated keypoint. Instead, it constrains the relative direction and distance of each keypoint pair in the student prediction, making the student keypoint set approach the stable 2D projection structure predicted by the clear-domain teacher. The pairwise weight is defined as

(11) $$q_{i}=v_{i}c_{i}^{\mathrm{illum}},\;w_{ij}=g_{T}\min\left(q_{i},q_{j}\right)I\left[\min\left(q_{i},q_{j}\right)\geq c_{0}\right],$$

where $v_{i}$ is keypoint visibility, $c_{i}^{\mathrm{illum}}$ is local illumination confidence, $g_{T}$ is the teacher gate based on Object Keypoint Similarity (OKS), and $c_{0}$ is the minimum confidence threshold. This weight emphasizes visible and reliable keypoint pairs; reduces interference from overexposed, shadowed, or severely degraded regions in structural distillation; and avoids forcing unreliable structures onto the student network.

Furthermore, a 3D-template weak-perspective reprojection loss is introduced to constrain the student prediction with the 3D rigid template. Let ${\tilde{u}}_{i}^{t}=\Pi_{\mathrm{wp}}\left(X_{i};A^{t},b^{t}\right)$ be the weak-perspective projected point fitted from the teacher keypoints. The loss is

(12) $$L_{3D}=\frac{\sum_{i}\alpha_{i}{∥u_{i}^{s}-{\tilde{u}}_{i}^{t}∥}_{1}}{\sum_{i}\alpha_{i}+\epsilon },\;\alpha_{i}=g_{T}v_{i}c_{i}^{\mathrm{illum}}.$$

The technical meaning of this loss is that the keypoint set output by the student network must be explainable as the 2D projection of the same 3D rigid template under the current viewpoint, rather than merely approaching teacher coordinates point by point. Pairwise distillation constrains the 2D relative topology, while the 3D reprojection loss further pulls this 2D structure back to the 3D template projection relation. Together, they constitute the core of topological-projection-consistent learning in this paper. Since the loss weights jointly consider the teacher gate, keypoint visibility, and illumination confidence, the network mainly transfers structural information from visible and geometrically trustworthy regions, thereby avoiding uncertain responses in extreme-illumination regions from corrupting the global structure.

The weak-perspective projection $\Pi_{\mathrm{wp}}$ is used only in the distillation regularizer $L_{3D}$; final pose estimation still uses the full perspective model in Section 3.3. For each training image, $\left(A^{t},b^{t}\right)$ is obtained by a closed-form least-squares fit to the teacher keypoints, providing a stable, non-iterative target for student training. Thus, $L_{3D}$ serves as a low-weight rigid-template regularizer rather than a pixel-exact replacement for perspective projection. Its approximation error is confined to the training loss, whereas the metric pose is recovered exclusively by full-perspective PnP. The approximation becomes less accurate for closer viewpoints or larger target depth variation.

The total loss is defined as

(13) $$\begin{array}{cc} L_{\mathrm{total}}= & \lambda_{\det}L_{\det}+\lambda_{\mathrm{cls}}L_{\mathrm{cls}}+\lambda_{\mathrm{kpt}}L_{\mathrm{kpt}}+\lambda_{3D}L_{3D}\\ \; & +\lambda_{\mathrm{pair}}L_{\mathrm{pair}}+\lambda_{\mathrm{query}}L_{\mathrm{query}}+\lambda_{\mathrm{feat}}L_{\mathrm{feat}}+\lambda_{\mathrm{rel}}L_{\mathrm{rel}}, \end{array}$$

where $L_{\det}$ is the hard-label detection loss; $L_{\mathrm{cls}}$, $L_{\mathrm{kpt}}$, $L_{\mathrm{query}}$, and $L_{\mathrm{feat}}$ align teacher–student outputs or features; $L_{\mathrm{pair}}$ and $L_{3D}$ impose topological-projection-consistent structural constraints; and $L_{\mathrm{rel}}$ supervises point-wise keypoint reliability. Dynamic OKS scale scheduling is used during training, while a fixed OKS scale is uniformly used during evaluation to ensure fair comparison.

The proposed formulation differs from conventional structural distillation in how the 2D relations are linked to the target geometry. Pairwise keypoint losses constrain relative 2D geometry within an image; $L_{\mathrm{pair}}$ has the same role and transfers the teacher’s view-specific keypoint configuration. However, this constraint alone does not require the predicted point set to belong to the projection family of the fixed 3D template under a valid camera transform. The additional $L_{3D}$ term provides this rigid-template projection anchor. In contrast, generic geometry-aware distillation commonly aligns class responses, features, or heatmaps without explicitly introducing a target-specific 3D template. The proposed topological projection consistency therefore arises from coupling 2D relational distillation with the 3D-template reprojection constraint.

### 3.3. Reliability–Reprojection-Residual Jointly Reweighted PnP

Standard PnP usually assumes that all 2D observations have the same noise level after RANSAC obtains the inlier set. However, in extreme-illumination images, different keypoints have different local visibility, texture quality, and topological consistency. Equal-weight optimization may give low-reliability keypoints excessive influence on the pose result. According to the Gauss–Markov criterion, this paper uses the network-predicted reliability as the observation-quality prior and the reprojection residual as the geometric posterior correction. For the inlier set $S_{\mathrm{in}}$, the weighted reprojection objective is

(14) $$\min_{R,t}\;\sum_{i\in S_{\mathrm{in}}}e_{i}^{⊤}W_{i}e_{i},\;e_{i}=u_{i}-\Pi \left(X_{i},K,d,R,t\right),$$

where $W_{i}$ is the information weight of the *i*-th observation. The reliability prior is defined as

(15) $$\omega_{\mathrm{pri},i}=\omega_{\min}+\left(1-\omega_{\min}\right)r_{i}^{\mathrm{kpt}},\;0<\omega_{\min}<1.$$

where $r_{i}^{\mathrm{kpt}}$ is the point-wise keypoint reliability, and $\omega_{\min}$ prevents low-reliability points that still have geometric contribution from being completely removed. This prior corresponds to an isotropic observation covariance

(16) $$\sigma_{i}=\frac{\tau }{\sqrt{\omega_{\mathrm{pri},i}}},\;\Sigma_{i}=\sigma_{i}^{2}I_{2},\;W_{i}=\Sigma_{i}^{-1}.$$

At the *k*-th iteration, the residual $e_{i}^{\left(k\right)}=u_{i}-\Pi \left(X_{i};K,d,R^{\left(k\right)},t^{\left(k\right)}\right)$ is used to compute $\eta_{i}^{\left(k\right)}={∥e_{i}^{\left(k\right)}∥}_{2}$, producing the geometric posterior weight

(17) $$\omega_{\mathrm{geo},i}^{\left(k\right)}={\mathrm{Proj}}_{\left[\omega_{\mathrm{geo},\min},1\right]}\left(\frac{1}{\sqrt{1+{\left(\eta_{i}^{\left(k\right)}/\tau_{r}\right)}^{2}}}\right),$$

where $\tau_{r}$ is the robust scale, and ${\mathrm{Proj}}_{\left[a,b\right]}\left(\cdot \right)$ denotes weight clipping. The joint weight is

(18) $$\omega_{\mathrm{joint},i}^{\left(k\right)}={\mathrm{Proj}}_{\left[\omega_{\mathrm{final},\min},1\right]}\left(\omega_{\mathrm{pri},i}^{\left(k\right)}\omega_{\mathrm{geo},i}^{\left(k\right)}\right),$$

The local pose-refinement objective is

(19) $$\underset{R,t}{\mathrm{arg min}}\sum_{i\in S_{\mathrm{in}}}\omega_{\mathrm{joint},i}^{\left(k\right)}∥u_{i}-\Pi \left(X_{i};K,d,R,t\right){∥}_{2}^{2}.$$

In implementation, RANSAC first provides the initial pose and inlier set, followed by several rounds of weighted least-squares refinement. This strategy does not simply remove low-confidence points. Instead, it jointly considers network reliability and reprojection residuals: low-reliability but projection-consistent points can still retain their geometric contribution, whereas high-confidence outliers with inconsistent reprojection are suppressed by residual-based weights. In this way, the backend PnP solver forms a closed loop with frontend topological-projection-consistent learning, so that the final pose is mainly determined by reliable and geometrically consistent 2D–3D correspondences.

The proposed pipeline also differs from PnP-based training and uncertainty formulations. End-to-end reprojection methods such as EPro-PnP differentiate through a full-perspective pose objective during training [30]; our frontend instead uses a closed-form weak-perspective target for distillation and applies perspective PnP only at inference. Uncertainty-aware PnP methods introduce per-point covariances in the backend [14,15], whereas our reliability $r_{i}^{\mathrm{kpt}}$ is learned jointly with the topological projection consistency losses and is subsequently combined with the reprojection residual. The distinction is therefore the coupling of projection-consistent supervision, structural feature learning, and reliability-aware pose refinement around the same 3D template.

## 4. Experiments

### 4.1. Experimental Setup and Dataset

This section verifies the proposed method from the perspective of topological projection consistency. The experiments do not only evaluate the average pixel error of 2D keypoints but also examine whether the keypoint set can provide stable 2D–3D correspondences for the backend PnP solver. The acquisition device is a monocular industrial camera with an image resolution of 3072 × 2160 and a lens focal length of 16 mm. The experimental target is a scaled satellite model with a maximum overall dimension of approximately 1 m. The measurement distance is approximately 1–5 m, corresponding to an equal-scale space measurement distance of approximately 3–15 m. To simulate close-range on-orbit observation, the target is fixed in front of a projection screen with a space background. Different degrees of extreme-illumination images are generated by changing exposure and active illumination intensity, as shown in Figure 6a. The target contains 17 semantic keypoints, including four points on each solar panel, eight points on the main body, and one antenna point for direction localization, as shown in Figure 6b.

The experimental data consist of three parts. The first part is real captured training data, including 583 target images under different degrees of extreme illumination, with 53 mildly degraded images and 530 severely degraded images. All images are annotated with 2D keypoints. The target structures in mildly degraded images are relatively clear and the annotations are more reliable, whereas the severely degraded images inevitably contain annotation uncertainty caused by highlight saturation, shadows, and texture loss.

The second part is projection-consistent augmented data rendered from mildly degraded images using the proposed data generation method. This part contains paired clear-domain and degraded-domain samples with known 2D keypoints, masks, and camera parameters. Clear-domain images are used to train the teacher network, and degraded-domain images are used for student-network distillation. Specifically, the teacher network is trained with 53 real mildly degraded images and 500 rendered clear-domain images, while the student network is trained with 530 real severely degraded images and 500 rendered degraded-domain images. Because the clear-domain and degraded-domain samples share their 3D template and projection relation, this part of the data is used to verify the effect of topological-projection-consistent supervision.

The third part is real test data. For keypoint evaluation, clear-reference images and corresponding degraded images are captured at fixed positions, and the annotations of the clear-reference images are used as the ground truth for the degraded images. For pose evaluation, the target and calibration target remain relatively fixed. The relation between the target coordinate system and the calibration-target coordinate system is obtained through reconstruction, and the ground-truth camera pose is obtained from the clear-reference calibration image. After removing samples with abnormal reconstruction or calibration quality, 320 valid extreme-illumination images are used for pose measurement. This setting ensures that all methods use the same camera parameters, 3D keypoint template, and pose-reference construction procedure, so that experimental differences mainly come from the frontend keypoint topology and backend optimization strategy.

The dataset combines complementary real and rendered data. The 53 mildly degraded and 530 severely degraded captures represent the degradation produced by the physical imaging setup, while the 500 clear-domain and 500 degraded-domain renders extend the viewpoint, orientation, and lighting coverage. Section 4.5 compares the photometric statistics of rendered, real degraded, and real normal images, and Section 4.9 evaluates the pipeline with three additional backgrounds. The 296-image keypoint test set and the 320-image pose test set are collected in independent sessions and do not reuse training images or rendered camera poses. This design broadens the evaluated conditions while retaining the target- and laboratory-specific limitations discussed in Section 5.

### 4.2. Evaluation Metrics and Compared Methods

The experiments include keypoint detection accuracy, pose measurement accuracy, and ablation analysis. Keypoint detection is evaluated using AP50, AP75, AP50:95, mean pixel error (MPE), and root mean square pixel error (RMSE). The AP metrics are calculated based on OKS, while MPE and RMSE directly measure the pixel distance between predicted and annotated keypoints. Considering that extreme illumination may cause a small number of completely incorrect keypoints and significantly increase the overall RMSE, this paper further separates normal localization errors from catastrophic errors. Keypoints with Euclidean pixel errors larger than 50 px are treated as outliers, and their number and ratio are reported. MPE_≤50_ and RMSE_≤50_ are computed on the remaining keypoints. This makes it clearer to distinguish “local localization accuracy” from “whether the topology is corrupted by outliers”.

For pose measurement, the 2D keypoints output by each detector are associated with the reconstructed 3D target keypoints in the calibration-target coordinate system. All methods use the same camera intrinsics, distortion parameters, 3D keypoint template, RANSAC parameters, and Levenberg–Marquardt (LM) reprojection refinement procedure. RANSAC independently obtains the initial pose and inlier set from the keypoints predicted by each method. The proposed reliability–reprojection-residual reweighted PnP then performs iterative weighted least-squares optimization using point-wise keypoint reliability and reprojection residuals. The pose metrics include rotation error, translation error, camera-center error, reprojection error, and the average number of inliers. Compared with 2D errors alone, these metrics directly reflect whether the keypoint set satisfies the global projection-geometry constraints required by PnP.

The compared methods include DETRPose [19], YOLOv11Pose [39], RTMPose [18], and HRNet [26]. No external pretrained weights are loaded for any method. All networks are trained using the AdamW optimizer with a batch size of 16. Except for the teacher network, which is trained for 500 epochs, all other networks are trained for 300 epochs.

The compared detectors cover four representative keypoint architectures: HRNet is a high-resolution CNN heatmap model, RTMPose is a lightweight top–down model with a SimCC-style head, YOLOv11Pose performs one-stage bounding-box and keypoint regression, and DETRPose uses Transformer queries for direct coordinate prediction.

DETRPose is selected as the teacher–student base for three reasons: its decoder exposes keypoint, query, and feature outputs required by the distillation terms in Equation (13); its fixed keypoint ordering corresponds directly to the 3D template indices; and it achieved the strongest one-stage AP among the evaluated baselines in our preliminary experiments. It therefore provides a competitive and structurally compatible foundation for the proposed method.

### 4.3. Data Augmentation Results

The data augmentation in this paper serves teacher-network and student-network training. Its core purpose is not to generate more visual appearances but to generate more paired training samples with consistent projection labels. The augmented data are generated from mildly degraded images, and the background images come from two groups of normally illuminated backgrounds. Because the number of mildly degraded images is small, target and scene rendering is implemented using Binocular3DGS [25], which is suitable for sparse data. Figure 7 shows the source images, background images, and rendered clear-domain and degraded-domain samples used for augmentation.

To verify the view consistency of the rendered clear-domain images, COLMAP [38] reconstruction is further performed on randomly selected rendered clear images. The recovered viewpoint trajectory and sparse point cloud in Figure 8 show that the rendered images preserve the perspective relation between the foreground target and the background, and can provide 3D constraints for teacher–student training. This verification is important because the augmented images are used as structural supervision rather than only for visual appearance diversification. If degraded rendering changes the apparent target contour while the projected keypoint labels no longer match the true camera geometry, the student network would learn contradictory image–keypoint correspondences. The reconstruction results show that the generated samples preserve view consistency at the image level, so the paired clear/degraded samples can transfer structural information from the teacher network to the student network more reliably.

### 4.4. Keypoint Detection Results

The keypoint detection experiment is divided into one-stage and two-stage settings. The one-stage methods directly train and test detectors on extreme-illumination images. The two-stage methods first process degraded images using an image restoration network and then train keypoint detectors. Both settings are evaluated to examine whether “improving visual appearance” is equivalent to “recovering topological-projection-consistent keypoints for PnP”. In the two-stage setting, the CSEC [11] exposure restoration network is used for image preprocessing, after which the same detection algorithms are trained and tested. A total of 296 test images are used for keypoint evaluation. Figure 9 shows representative detection results, and Table 1 summarizes the results on all extreme-illumination images. Bold and underlined values indicate the best and second-best results, respectively.

On all extreme-illumination test images, the proposed method achieves the highest AP values, the lowest inlier pixel errors, and the fewest outlier keypoints. Compared with the strongest one-stage baseline, DETRPose, the proposed method increases AP50 from 0.918 to 0.990, AP75 from 0.480 to 0.867, and AP50:95 from 0.555 to 0.737. At the same time, MPE_≤50_ decreases from 19.98 px to 15.37 px, RMSE_≤50_ decreases from 23.51 px to 18.96 px, and the number of outlier keypoints decreases from 934 (21.0%) to 407 (9.2%). The improvements in AP75 and AP50:95 are much larger than that in AP50, indicating that the compared methods can usually locate the target coarsely but cannot stably preserve the keypoint structure under stricter localization thresholds. For PnP, a small number of topologically incorrect or semantically mismatched 2D–3D correspondences can significantly distort the final pose. Therefore, the outlier ratio and strict-threshold AP better reflect the projection-geometric reliability of the keypoint set.

The outlier-separated metrics further reveal the error characteristics of the different methods. YOLOv11Pose has a competitive AP50 and moderate inlier error, but 31.6% of its keypoints have errors larger than 50 px, indicating frequent semantic mismatches or structural jumps. RTMPose is more unstable, with an outlier-keypoint ratio of 44.6% and an AP75 of only 0.008. In contrast, the proposed method simultaneously maintains a high AP75, low inlier error, and the lowest outlier ratio. This indicates that projection-consistent supervision and topological-projection-consistent distillation improve not only keypoint recall but also semantic localization and global structural stability. Table 2 further reports the detection results on mildly degraded images.

All methods improve on mildly degraded images, confirming that extreme illumination is a major factor that corrupts keypoint localization and structural consistency. Even when degradation is weaker, the proposed method still maintains a clear advantage. Compared with DETRPose, AP75 increases from 0.524 to 0.899, AP50:95 increases from 0.582 to 0.779, and MPE decreases from 38.48 px to 20.91 px. This shows that the proposed method does not merely handle extreme failure cases but also improves fine-grained localization through topological projection consistency constraints when target structures are partially visible.

In addition, Table 1 shows that the image-restoration-based two-stage setting does not provide stable improvement. For all four compared detectors, the AP50:95 after CSEC preprocessing is lower than that of the corresponding one-stage method. For example, DETRPose decreases from 0.555 to 0.425 in AP50:95, and its outlier-keypoint ratio increases from 21.0% to 28.7%. This indicates that visually enhanced images are not necessarily better measurement images. The reason is that image restoration mainly optimizes appearance quality, whereas keypoint–PnP pose measurement requires restored contours and corners to remain consistent with their true projected positions. The CSEC processing result is shown in Figure 10.

In most images used in this study, strong reflection makes local regions completely white. CSEC-type methods can restore mildly overexposed scenes to some extent, but the true target textures in fully saturated regions have already been lost. The restoration network can only infer missing structures from image priors. These inferred structures may appear visually reasonable, but their edges and corners do not necessarily coincide with true projected keypoints. Training detectors on such restored images may introduce artificial textures, edge displacement, or oversmoothed boundaries, further corrupting topological projection consistency. Therefore, treating extreme illumination simply as an image-restoration problem is insufficient for geometric measurement.

It should be noted that RTMPose has a very low AP75 under both illumination conditions. The experimental target has weak texture, similar local appearances, and low contrast with the background, making RTMPose less suitable for detecting solar-panel edge keypoints. Edge keypoints lie on low-texture boundaries and are visually difficult to localize, but they provide a large spatial baseline for PnP and have a high geometric contribution. YOLOv11Pose also produces many unavoidable large errors on edge points. Representative abnormal cases are shown in Figure 11. These outliers may be removed by RANSAC, but their presence reduces the number of stable correspondences and weakens the 3D template projection constraint. Therefore, robust space-target pose measurement requires keypoints with stable topological projection consistency.

### 4.5. Sim-to-Real Distribution Validation

To assess whether the rendered data provide relevant supervision for keypoint learning, we compare 500 rendered degraded training images with 222 real degraded images and 74 real normal-illumination images acquired using the same target and laboratory setup. All statistics are computed within a common padded bounding box defined by the 17 labelled keypoints, because identical alpha masks were unavailable for the real images.

The reported indicators are mean luminance, near-saturated pixel ratio (I≥0.95), dark-pixel ratio (I≤0.05), specular-highlight proxy area (I≥0.92 and RGB chroma ≤0.08), and mean gradient magnitude. We additionally compute the Jensen–Shannon (JS) divergence between luminance histograms and the *z*-normalized distance between mean photometric feature vectors, both relative to the real degraded pool.

As shown in Table 3, the rendered images exhibit substantially stronger overexposure than the real degraded images: their mean luminance, saturated-pixel ratio, and specular-proxy ratio are 0.695, 23.85%, and 19.70%, compared with 0.311, 5.60%, and 4.90% for the real degraded pool. They should therefore be interpreted as deliberately strong photometric stress cases rather than as samples from an identical distribution. The real normal pool has a smaller luminance-histogram JS divergence, whereas the rendered pool is slightly closer in the combined photometric feature space (1.140 versus 1.271). Taken together, these results show that the rendering reproduces and extends the highlight and saturation cues relevant to extreme-illumination keypoint training, while not establishing strict synthetic-to-real distributional equivalence.

### 4.6. Pose Measurement Experiment

The ground truth of the pose measurement images is obtained from a calibration target fixed below the object, and the relative position between the object and calibration target remains unchanged. The clear-reference calibration image provides the ground-truth camera pose, and the 3D target keypoints are reconstructed from manually annotated 2D observations in the calibration-target coordinate system. After removing samples with abnormal reconstruction or calibration quality, 320 extreme-illumination images are used for pose measurement. This setting ensures that all methods are evaluated with the same camera parameters, 3D template, and pose-reference construction process. Therefore, differences in pose results mainly come from the topological projection consistency of the frontend predicted keypoint set and the backend optimization strategy. Figure 12 shows pose-test samples, and Figure 13 visualizes the ground-truth and measured camera positions from 50 randomly selected viewpoints.

Table 4 reports the pose estimation results from all the test images. For the proposed detector, both direct PnP and reliability–reprojection-residual reweighted PnP are evaluated. All methods use the same RANSAC algorithm and parameter settings, but each method independently determines its initial pose and inlier set according to its own predicted keypoints. Subsequent refinement and final inlier statistics are also performed independently.

Table 4 shows that the improvement in pose accuracy comes from both frontend topologically consistent keypoint detection and backend reliability weighting. First, Ours-DirectPnP already outperforms all compared methods in rotation, translation, and camera-center errors, indicating that the proposed teacher–student keypoint network provides more stable 2D–3D correspondences under extreme illumination. Compared with DETRPose, Ours-DirectPnP reduces rotation RMSE from 4.58° to 2.05°, translation RMSE from 48.94 mm to 31.16 mm, and camera-center RMSE from 219.26 mm to 94.97 mm. This indicates that improving the topological projection consistency of the frontend keypoint set is the main source of the final pose accuracy gain.

Second, the comparison between Ours-DirectPnP and Ours-rel-PnP reflects the effect of reliability–reprojection-residual reweighting. Ours-rel-PnP reduces the translation mean error from 21.93 mm to 21.17 mm and the translation RMSE from 31.16 mm to 29.49 mm, while increasing the average number of inliers from 15.92 to 16.56. This shows that predicted keypoint reliability and residual correction can preserve geometrically useful observations and reduce the influence of low-quality or projection-inconsistent points. It should be noted that reweighting does not simply pursue the minimum unweighted reprojection error. Instead, it changes the contribution of each point to pose estimation according to observation reliability and geometric residuals, so it is reasonable that some rotation-related metrics are very close to DirectPnP.

Compared with the DETRPose baseline, the final proposed method reduces rotation RMSE, translation RMSE, and camera-center RMSE by approximately 55.1%, 39.8%, and 56.3%, respectively. The table also shows that a smaller reprojection error does not necessarily imply a more accurate spatial pose. YOLOv11Pose obtains the smallest reprojection RMSE, but its rotation, translation, and camera-center errors are much larger than those of the proposed method. This occurs because reprojection error measures the internal consistency between the estimated pose and detected 2D points. If the detected keypoints themselves have systematic bias or semantic mismatch, the reprojection error may still be small, while the recovered 3D pose deviates from the ground truth. Therefore, for PnP-oriented space-target keypoint detection, spatial pose errors and inlier statistics better reflect topological projection consistency than reprojection error alone.

Figure 14 further supports the numerical results in Table 4. The proposed method has the most concentrated distributions of rotation, translation, and camera-center errors, with obviously fewer long-tail errors and abnormal poses than the compared algorithms. This is particularly important for extreme-illumination tasks, because strong illumination usually corrupts only local regions of the target, but a small number of unreliable keypoints can cause large pose deviations in PnP. The proposed method reduces structural keypoint errors through frontend topological-projection-consistent learning and reduces the influence of unreliable observations through backend reliability weighting, thereby suppressing the long tail of pose errors.

### 4.7. Proxy-Geometry Sensitivity

Because the proxy P supplies illumination and visibility cues rather than an exact surface model, we evaluate the effect of controlled errors in its non-keypoint vertices. An absolute Chamfer distance cannot be reported because no independent CAD model or surface scan of the physical target is available. Instead, the surface deviation ratio η is defined as the maximum normal displacement relative to the body-keypoint bounding-box diagonal *D*, as follows:

(20) $$v_{j}^{\left(\eta \right)}=v_{j}+\eta D\;s_{j}n_{j},$$

where $n_{j}$ is the local normal and $s_{j}$ is a smooth zero-mean field with $|s_{j}|\leq 1$. The field is generated by Laplacian-type smoothing while all semantic-keypoint vertices remain fixed. Consequently, the 2D labels and 3D PnP template are unchanged; only the rendered reflection, saturation, and approximate visibility patterns vary.

We evaluate η∈{0.005,0.010,0.020}. With D≃0.780 m, these levels correspond to maximum displacements of 3.90, 7.80, and 15.60 mm. One fixed perturbation realization is used at each level. For every condition, a new degraded training set is rendered and a student is trained for 300 epochs from the same frozen teacher, using the main-experiment optimizer, batch size, random seed, camera poses, backgrounds, and lighting seed. Evaluation uses the same keypoint and 320-image pose test sets, with DirectPnP applied to all conditions. The experiment is a controlled single-realization sensitivity analysis rather than a statistical study across perturbation seeds. The effect of the illumination difference generated by different disturbances is shown in Figure 15, where both the panel and the main body present different degrees of illumination difference. The specific quantization results are shown in Table 5.

As shown in Table 5, the two smaller perturbations produce only minor changes in AP75 and AP50:95, but increase camera-center RMSE from 94.97 mm to 113.82 and 128.23 mm. At η=0.020, AP75 decreases to 0.817 and the outlier ratio rises to 11.60%. The pose errors are not monotonic because each level uses a different single perturbation field. The results therefore support a limited conclusion: keypoint detection tolerates moderate proxy deviations, whereas pose accuracy is more sensitive to the resulting reflection and visibility changes. Repeated perturbation seeds and independent surface ground truth would be required for statistical or absolute proxy-fidelity assessment.

### 4.8. Ablation Study

To analyze the contribution of each component to topological projection consistency and pose-solving, ablation experiments are conducted on the same degraded keypoint test set and the same 320 pose measurement images. Five variants are evaluated. Baseline denotes the original DETRPose without the proposed topological-projection-consistent augmentation (TPCA), reliability-aware network, structural distillation, or reliability–reprojection-residual reweighted PnP. TPCA only uses the same plain DETRPose architecture as Baseline, but is trained with topological-projection-consistent degraded-domain augmented samples; therefore, it evaluates the data-level contribution of topological-projection-consistent supervision. w/o RG-CCEM keeps the reliability-aware distillation framework and complete loss terms, but removes the global structural propagation module. w/o SD keeps the full network structure, reliability supervision, and basic classification/keypoint distillation, but disables structural-distillation-related terms, including pairwise structural distillation, 3D reprojection distillation, query distillation, and feature distillation. Full denotes the complete detector and uses the final checkpoint consistent with One-stage Ours in the main experiments. The pose columns in Table 6 all use the same DirectPnP backend for fair comparison of the geometric usability of frontend keypoint sets.

Table 6 shows that TPCA brings the largest 2D keypoint improvement over the original detector. Compared with Baseline, TPCA only increases AP75 from 0.480 to 0.847 and AP50:95 from 0.555 to 0.755, while reducing MPE_≤50_ from 19.98 px to 14.79 px, RMSE_≤50_ from 23.51 px to 18.72 px, and the outlier-keypoint ratio from 21.0% to 8.6%. The corresponding pose errors are also substantially reduced, with rotation RMSE decreasing from 4.58° to 3.33°, translation RMSE from 48.94 mm to 42.87 mm, and camera-center RMSE from 219.26 mm to 149.26 mm. This indicates that topological-projection-consistent augmentation does not merely increase the number of training samples. More importantly, it provides degraded-domain supervision that is consistent with camera geometry and the 3D target template.

The comparison among TPCA only, w/o RG-CCEM, w/o SD, and Full further demonstrates the effects of the network structure and distillation constraints. Compared with w/o RG-CCEM and w/o SD, Full obtains higher AP75, lower MPE_≤50_ and RMSE_≤50_, and the lowest DirectPnP pose errors. When RG-CCEM is removed, rotation RMSE, translation RMSE, and camera-center RMSE increase from 2.05°, 31.16 mm, and 94.97 mm to 2.59°, 43.09 mm, and 122.50 mm, respectively. This shows that global structural propagation helps recover pose-relevant target topology when local features are degraded. When structural distillation is disabled, the pose errors also increase to 2.34°, 38.66 mm, and 108.73 mm, indicating that pairwise structural constraints and 3D reprojection constraints indeed improve the spatial consistency of the keypoint set.

It should be noted that TPCA only is slightly better than Full on several unweighted 2D pixel-error metrics. This does not negate the effectiveness of the proposed modules. Instead, it supports the central argument of this paper: pose measurement accuracy is not completely determined by keypoint extraction accuracy alone; the key issue is that the 2D topology of the extracted keypoints must maintain a consistent topological projection relation with the 3D template points. TPCA only is a single-task 2D keypoint localizer, and all model capacity and loss weights serve pixel coordinate regression. It is therefore a strong reference for absolute 2D localization accuracy. Full, in contrast, is a multi-objective model for pose measurement. In addition to coordinate regression, it learns reliability, teacher–student feature alignment, pairwise structural relations, and 3D template projection constraints. These objectives are not always directly rewarded by average pixel error, but they are critical for selecting and constraining the 2D–3D correspondences required by PnP. Therefore, the small 2D error gap between TPCA only and Full should be interpreted together with the pose results and topological projection consistency, rather than simply as a failure of RG-CCEM or structural distillation.

To avoid repeating Table 6, Table 7 gives a compact diagnostic comparison between TPCA only and Full. The pose-image statistics are reported on the 319 images on which both TPCA only and Full successfully return a pose estimate; one Full sample fails in DirectPnP and is excluded from the paired image-level comparison.

These diagnostic results support a more precise conclusion. TPCA mainly explains the large 2D localization improvement over the original DETRPose baseline. On this basis, RG-CCEM and structural distillation further improve the geometric usability of the network compared with their respective ablated variants. The apparent difference between TPCA only and Full on the independent keypoint test set is small: MPE_≤50_ differs by only 0.58 px, RMSE_≤50_ by only 0.24 px, and the outlier ratio by only 0.6 percentage points. This difference may be related to data splitting and multi-objective training tradeoffs. However, on the pose measurement subset, Full provides lower common-keypoint errors and achieves better pose results on most images. Therefore, 2D keypoint accuracy is a necessary but insufficient condition for high-accuracy pose measurement; the spatial consistency, reliability, and geometric contribution of the keypoint set also determine the final PnP result.

Because TPCA only does not predict keypoint reliability, the complete reliability–reprojection-residual reweighted PnP used by Full cannot be directly applied to it without adding an additional reliability estimator. To exclude the possibility that the pose improvement is mainly caused by adding a second-stage weighted PnP backend, an additional backend-control experiment is conducted. In this experiment, the reliability priors of both TPCA only and Full are fixed to 1, so the backend refinement degenerates into residual-only iteratively reweighted least squares (IRLS). Therefore, residual-only IRLS uses only the reprojection residual as a posterior geometric weight and does not use the learned image-domain keypoint reliability. This setting gives TPCA only and Full the same residual-based geometric reweighting opportunity, allowing the contribution of the frontend keypoint set to be isolated. The results are shown in Table 8.

Table 8 shows that applying the same residual-only IRLS backend to TPCA only slightly reduces its translation RMSE and camera-center RMSE from 42.87 mm and 149.26 mm to 40.77 mm and 146.65 mm, respectively. These results remain clearly worse than the 31.16 mm and 94.97 mm obtained by Full + DirectPnP. Therefore, the pose advantage of Full is not caused only by residual-based backend refinement. Even when TPCA only is given the same residual-only reweighting opportunity, the gap with Full cannot be closed. This indicates that the keypoint set output by the Full frontend itself has better topological projection consistency and PnP solvability.

It should also be noted that residual-only IRLS can obtain strong results when the initial pose and inlier set are already reliable, because the reprojection residual directly reflects posterior projection consistency under the current pose hypothesis. However, residual weighting is still dependent on the initial solution and cannot describe image-domain observation quality before pose refinement. The learned keypoint reliability instead provides a prior related to local illumination degradation, texture loss, and feature ambiguity. Therefore, the role of reliability–reprojection-residual reweighted PnP is to combine image-domain reliability priors with projection-domain residual corrections, rather than to guarantee metric-wise dominance over residual-only IRLS in every evaluation item. In the main experiment, this joint weighting reduces the translation RMSE to 29.49 mm and increases the average number of inliers. Together with the backend-control results, the experiments show that the proposed method improves pose measurement through a coupled pipeline: TPCA provides topological-projection-consistent degraded-domain supervision; RG-CCEM and structural distillation improve PnP-relevant feature and keypoint topology consistency; and the backend PnP solver uses reliability and residuals to reduce the influence of unreliable or projection-inconsistent observations.

### 4.9. Cross-Background Evaluation

To evaluate the pipeline under different background environments, we construct an additional dataset with three visually and geometrically distinct environments (background_1, background_2, and background_3). The physical target, 3D keypoint template, proxy geometry $P_{0}$, and 17-keypoint definition are kept unchanged so that the influence of the background can be evaluated separately.

A background-specific 3DGS model is reconstructed for each environment. The resulting clear–degraded rendered pairs from the three environments are merged into a single training corpus. Unlike the main experiment, the teacher and student networks are both retrained for this experiment. A new teacher is first trained on the merged clear-domain data, and the student is subsequently trained on the corresponding degraded-domain data using this newly trained teacher. The training protocol, optimizer settings, epoch numbers, and random seed are kept consistent with those of the main experiment. Thus, the teacher is fixed only during the student distillation stage and is not reused from the main experiment.

For testing, each real extreme-illumination image is paired with a corresponding ideal-illumination image captured from the same viewpoint. The ideal-illumination images are annotated using the same 17-keypoint definition, and their annotations are used as the ground truth for the corresponding extreme-illumination images. Therefore, the degraded test images are not independently annotated. This experiment evaluates the adaptability of the complete pipeline after background-specific reconstruction and retraining, rather than the zero-shot transfer of the student trained in the main-scene experiment.

Only the background is varied; the target geometry, surface material, proxy topology, and degradation formulation remain fixed. We report AP50, AP75, AP50:95, MPE_≤50_, RMSE_≤50_, and the 50-pixel outlier ratio on the aggregated test set. Representative visualization results are shown in Figure 16, and the comparison results with the DETRPose baseline are presented in Table 9.

As shown in Table 9, DETRPose achieves a higher AP50 than the proposed method (0.9540 versus 0.9299), whereas the proposed method performs better under stricter localization criteria. AP75 increases from 0.2357 to 0.4471, AP50:95 increases from 0.4025 to 0.4746, and the outlier ratio decreases from 41.83% to 30.37%. MPE_≤50_ and RMSE_≤50_ are also reduced to 21.53 and 24.94 px, respectively. These results indicate that the structural constraints remain beneficial when the pipeline is retrained with varied background environments, particularly for precise localization and outlier suppression.

This experiment changes only the background and therefore does not establish generalization to new target geometries or surface materials. Such evaluation would require a new 3D template or proxy configuration and remains a subject for future work, as discussed in Section 5.

### 4.10. Hyperparameter Sensitivity Analysis

We conduct a one-factor-at-a-time analysis of the three principal training weights, $\lambda_{\mathrm{pair}}$, $\lambda_{3D}$, and $\lambda_{\mathrm{rel}}$, and the two backend parameters, $\tau_{r}$ and $\omega_{\min}$. Their default values are 0.08, 0.02, 0.35, 8 px, and 0.2, respectively. Each parameter is evaluated at 0.5× and 2× its default while the others remain fixed. To isolate the contribution of the 3D reprojection term, we additionally retrain the student with $\lambda_{3D}=0$, keeping the architecture, data, frozen teacher, all other loss weights, optimizer, effective batch size, random seed, and training schedule unchanged. The training-weight variants require a 300-epoch student retraining, whereas the backend parameters are evaluated on unchanged keypoint predictions. The frontend metrics use the complete 296-image keypoint test set. For the pose-related metrics, we exclude the single sample with extremely large estimation errors under some parameter configurations. To ensure fair comparisons, this sample is removed across all experimental settings, leaving a subset of 319 valid images for evaluation. All pose estimations adopt the reliability–reprojection-residual reweighted PnP backend. The detailed results are shown in Table 10.

The isolated comparison in Table 10 directly quantifies the contribution of $L_{3D}$ while all other proposed components remain active. Disabling this term decreases AP75 from 0.867 to 0.795 and increases the 50-pixel outlier ratio from 9.17% to 12.00%. On the matched 319-image pose subset, it also increases rotation, translation, and camera-center RMSE from 2.04°, 29.38 mm, and 95.54 mm to 3.01°, 35.40 mm, and 149.76 mm, respectively. Equivalently, enabling the default 3D term reduces these three pose RMSEs by 32.2%, 17.0%, and 36.2% relative to the isolated ablation. These results indicate that the weak 3D reprojection constraint reduces gross keypoint mismatches and improves the geometric usability of the predicted point set for PnP.

The omitted pose sample is not discarded as a data-quality exception. With $\lambda_{3D}=0$, this failure sample yields a geometrically incorrect keypoint configuration and a catastrophic PnP solution: the reliability-reweighted result has rotation, translation, and camera-center errors of 30.78°, 17,696.33 mm, and 17,749.15 mm, respectively. DirectPnP yields the same approximately 17.7 m failure, confirming that the error originates from the ablated frontend rather than the reliability-reweighted refinement. If this failed pose is included, the full 320-image RMSEs for $\lambda_{3D}=0$ become 3.46°, 989.89 mm, and 1003.41 mm. We therefore explicitly treat this case as a single catastrophic failure and adopt the same remaining 319 images for all rows in the finite-error comparison. This avoids letting one outlier solution dominate the RMSE metric, as well as preventing selectively adjusting the evaluation subset only for a single experimental setting.

Among the positive $\lambda_{3D}$ values, the default 0.02 provides the best overall pose accuracy. Increasing it to 2× raises rotation, translation, and camera-center RMSE to 3.51°, 71.16 mm, and 173.77 mm, respectively, showing that the 3D term should remain a weak regularizer rather than dominating the supervised keypoint objective. The other positive loss-weight perturbations keep AP75 between 0.830 and 0.874 and the outlier ratio between 8.60% and 10.00%. By contrast, the backend-only settings remain stable: their rotation RMSE ranges from 2.03° to 2.07° and their camera-center RMSE from 94.82 to 96.78 mm. Thus, the isolated ablation supports the contribution of $L_{3D}$, while the complete sensitivity analysis supports the selected small default weight and shows that the result is not an artifact of a narrowly tuned PnP backend.

### 4.11. Topological Projection Consistency Metric and Statistical Significance

To quantify agreement with the rigid 3D template, we evaluate all detectors on the pose test set using a topological projection consistency score. Given the reference pose $\left(R^{*},t^{*}\right)$, the projected template defines a rigid 2D reference. For predicted keypoints ${\left\{u_{i}\right\}}_{i=1}^{N}$, we compute

(21) $${\mathrm{TPC}}_{\mathrm{GT}}=\sqrt{\frac{1}{N}\sum_{i=1}^{N}\|u_{i}-\Pi \left(X_{i},K,d,R^{*},t^{*}\right){\|}_{2}^{2}.}$$

Unlike annotation-based MPE and RMSE, ${\mathrm{TPC}}_{\mathrm{GT}}$ uses the projection of the fixed 3D template under the reference pose. It therefore measures agreement with a rigid template projection, although it still contains an absolute keypoint-localization component. We also report ${\mathrm{TPC}}_{\mathrm{Proc}}$, the residual after fitting an optimal 2D similarity transform between the prediction and the projected template.

For each baseline, Table 11 reports the paired two-sided *t*-test for per-image ${\mathrm{TPC}}_{\mathrm{GT}}$ relative to our method. For the principal DETRPose comparison, the following analysis additionally reports 95% bootstrap confidence intervals over 10,000 resamples for the TPC and pose-error improvements.

Our method obtains the lowest ${\mathrm{TPC}}_{\mathrm{GT}}$ and ${\mathrm{TPC}}_{\mathrm{Proc}}$, reducing them relative to DETRPose from 30.80 to 15.20 px and from 27.91 to 11.38 px, respectively. MPE_≤50_, RMSE_≤50_, and the outlier ratio show the same trend.

Against DETRPose, the mean paired improvement in ${\mathrm{TPC}}_{\mathrm{GT}}$ is 15.60 px (95% confidence interval: [13.94, 17.27] px; $p<10^{-4}$), and the improvement in ${\mathrm{TPC}}_{\mathrm{Proc}}$ is 16.53 px ([15.29, 17.78] px; $p<10^{-4}$). The paired rotation-, translation-, and center-error improvements are 1.572° ($p<10^{-4}$), 5.17 mm (p=0.0294), and 75.08 mm ($p<10^{-4}$), respectively. These results indicate that the improvement is consistent across the paired test images rather than being determined by a small number of samples.

### 4.12. rel-PnP Stress Analysis

Because DirectPnP, residual-only IRLS, and rel-PnP have similar average performances in the main experiment, we compare them on subsets of the 320-image pose test set using the same Full-student predictions. The first difficulty axis is the number of valid keypoints, grouped as $n_{\mathrm{valid}}\leq 14$, $15\leq n_{\mathrm{valid}}\leq 16$, and $n_{\mathrm{valid}}=17$. The second is initialization quality: images in the worst 25% by initial RANSAC mean reprojection residual are assigned to the poor-initialization subset, with a threshold of 7.136 px. In the tables, *N* denotes successful pose estimates over the number of images in each subset.

As shown in Table 12, rel-PnP performs best across all three errors when 15–16 keypoints are valid, while remaining comparable to residual-only IRLS when all 17 points are available. Table 13 further shows that rel-PnP gives the lowest rotation and translation RMSE in the poor-initialization subset. However, when at most 14 points are valid, rel-PnP achieves one additional successful estimate but produces larger errors; residual-only IRLS is also better in the well-initialized subset. Though rel-PnP is motivated by the Gauss–Markov theorem, neither the learned keypoint reliability prior nor iteratively updated reprojection residuals yield strictly accurate normalized observation variances; they serve only as approximate surrogates for true noise statistics. Such approximation errors can limit performance gains and may even induce suboptimal outcomes under harsh observation conditions. The learned reliability prior is therefore most useful for moderately incomplete observations or imperfect initialization, and should be regarded as a complementary refinement mechanism rather than a universally superior backend.

### 4.13. Model Complexity and Runtime

We report the complexity, inference speed, and training time of the DETRPose-family models used in the proposed pipeline. Parameters, MACs, and FLOPs are measured at the main-experiment input resolution. Inference is evaluated on one NVIDIA TITAN V with batch size one, 400 warm-up iterations, and 1000 timing iterations. Training time is taken from the runs that produced the reported checkpoints. Embedded space-qualified hardware was unavailable; the TITAN V measurements therefore provide a desktop-GPU reference.

As shown in Table 14, only the active student path is included in deployment complexity; the frozen teacher stored in the training checkpoint is not executed at inference. Relative to DETRPose, the student increases parameters by 10.0% and FLOPs by 14.0%. Latency rises from 29.28 to 48.48 ms, reducing throughput from 34.15 to 20.63 FPS. The teacher and baseline share the same inference architecture and therefore have identical measured complexity, while their wall-clock training times differ due to inherent random fluctuations during model training. The added modules thus incur a measurable cost, although the student remains usable at approximately 20 FPS on the tested GPU. Pruning, low-precision inference, and module fusion are possible deployment optimizations but are not evaluated here. The runtime exhibits random fluctuations subject to hardware conditions, which do not affect the conclusions drawn from the experimental results.

## 5. Discussion

The experimental results show that the difficulty caused by extreme illumination is not merely a decrease in image quality but the destruction of topological projection consistency in the space-target keypoint set. For keypoint–PnP pose measurement, the decisive factor is whether the predicted 2D keypoints can be jointly explained as the projection of the same 3D rigid target under a single camera pose. Strong reflection, saturation, and shadows can remove true local evidence, generate false edges, or shift apparent contours, causing keypoint errors to become spatially nonuniform. In this case, even if some individual points appear accurately localized, the whole keypoint set may still fail to satisfy a consistent 2D–3D projection relation. Once these observations enter PnP, the backend optimization must compromise among mutually conflicting geometric constraints, thereby producing long-tailed pose errors.

This perspective also explains why image enhancement methods are insufficient for this problem. A visually clearer image is not necessarily a geometrically more reliable measurement image. When the true edges and corners in saturated regions have already been lost during imaging, a restoration network can only infer missing structures from image priors. The restored textures may improve visual quality, but their edge and corner positions may still deviate from the true projected positions. Therefore, enhancement-before-detection methods may reduce apparent degradation, but cannot restore the rigid projection consistency required by PnP.

The proposed method improves pose accuracy because it constrains this consistency at three coupled levels. At the data level, projection-consistent augmentation generates degraded-sample labels from the same camera pose, target template, and visibility relation. At the network level, teacher–student distillation transfers not only individual point coordinates but also pairwise structures and 3D template projection relations, reducing the possibility that the student network learns mutually incompatible keypoints. At the backend level, reliability–reprojection-residual reweighted PnP reduces the influence of observations that are unreliable in the image domain or inconsistent in the projection domain. Therefore, the results of this paper should be understood as pose improvements brought by maintaining valid 2D–3D correspondence geometry throughout the whole measurement pipeline, rather than as the effect of any single visual enhancement module.

This study is still limited by its available experimental conditions. Validation is performed in a laboratory-based semi-physical environment with limited target models, surface materials, cameras, backgrounds, and illumination ranges. In addition, the reference pose is obtained through camera calibration, manual 2D observations, and 3D keypoint reconstruction, and therefore still contains a certain degree of uncertainty. Future work will test more target structures and reflective materials, introduce a more independent pose reference system, and evaluate absolute measurement accuracy and cross-scene generalization under broader extreme-illumination conditions.

The cross-background experiment provides evidence only for adaptation to varied backgrounds after reconstruction and retraining. Generalization to target geometry remains untested: a new target requires a corresponding 3D keypoint template and proxy topology. Changes in solar-panel proportions or non-convex body geometry may particularly affect the pairwise and weak-perspective constraints. Surface materials are also represented only through the reflection, saturation, halo, and shadow modes included in D; substantially different optical responses would require additional degradation parameters. The current illumination setup uses active illumination and a fixed sun-simulator direction, without multi-source or dynamic illumination.

The reference pose is reconstructed from calibrated observations rather than measured by an independent metrology system. Its calibration and reconstruction uncertainty therefore introduces a common error floor for all methods. Future work will evaluate additional target geometries, materials, and illumination configurations, as well as an external pose reference.

The reported results use a 3072 × 2160 camera, a 16 mm lens, and a 1–5 m measurement range. Image-plane metrics depend primarily on the target’s pixel scale and feature visibility. Changing the camera therefore requires updated intrinsic and distortion calibration and may also require adjustment of the OKS scale and reprojection thresholds.

Metric translation and camera-center errors vary with focal length, range, and target size: configurations that produce a larger target projection generally provide finer spatial sensitivity. The weak-perspective regularizer also becomes less accurate at close range or when target depth variation is large. Consequently, the millimeter-level errors reported here should not be transferred directly to substantially different imaging geometries.

If the target occupies a markedly different fraction of the image or the sensor introduces a new photometric domain, retraining or fine-tuning is advisable. The 3D-template constraints and perspective PnP formulation remain applicable after camera recalibration.

## 6. Conclusions

This paper proposes a space-target pose measurement method based on feature-level topological projection consistency under extreme illumination. The core argument is that high-accuracy keypoint–PnP pose-solving depends on a topological-projection-consistent 2D keypoint set, rather than only on image clarity or low average individual point error. To maintain this consistency, the proposed method first generates clear–degraded paired samples with shared projection labels. Then, through RG-CCEM, pairwise structural distillation, and 3D-template weak-perspective reprojection constraints, the rigid keypoint topology learned by the clear-domain teacher network is transferred to the degraded-domain student network. Finally, predicted keypoint reliability and reprojection residuals are used for PnP weighted optimization. Compared with the DETRPose baseline, the proposed method reduces MPE_≤50_ and RMSE_≤50_ by approximately 23.1% and 19.4%, decreases the outlier-keypoint ratio from 21.0% to 9.2%, and reduces rotation, translation, and camera-center RMSE by approximately 55.1%, 39.8%, and 56.3%, respectively. The experimental results demonstrate that explicitly preserving consistency between the 2D feature topology and the projected 3D template topology from data generation to network learning and backend optimization is an effective way to improve the robustness and accuracy of space-target pose measurement under extreme illumination.

## Figures and Tables

**Figure 1:**
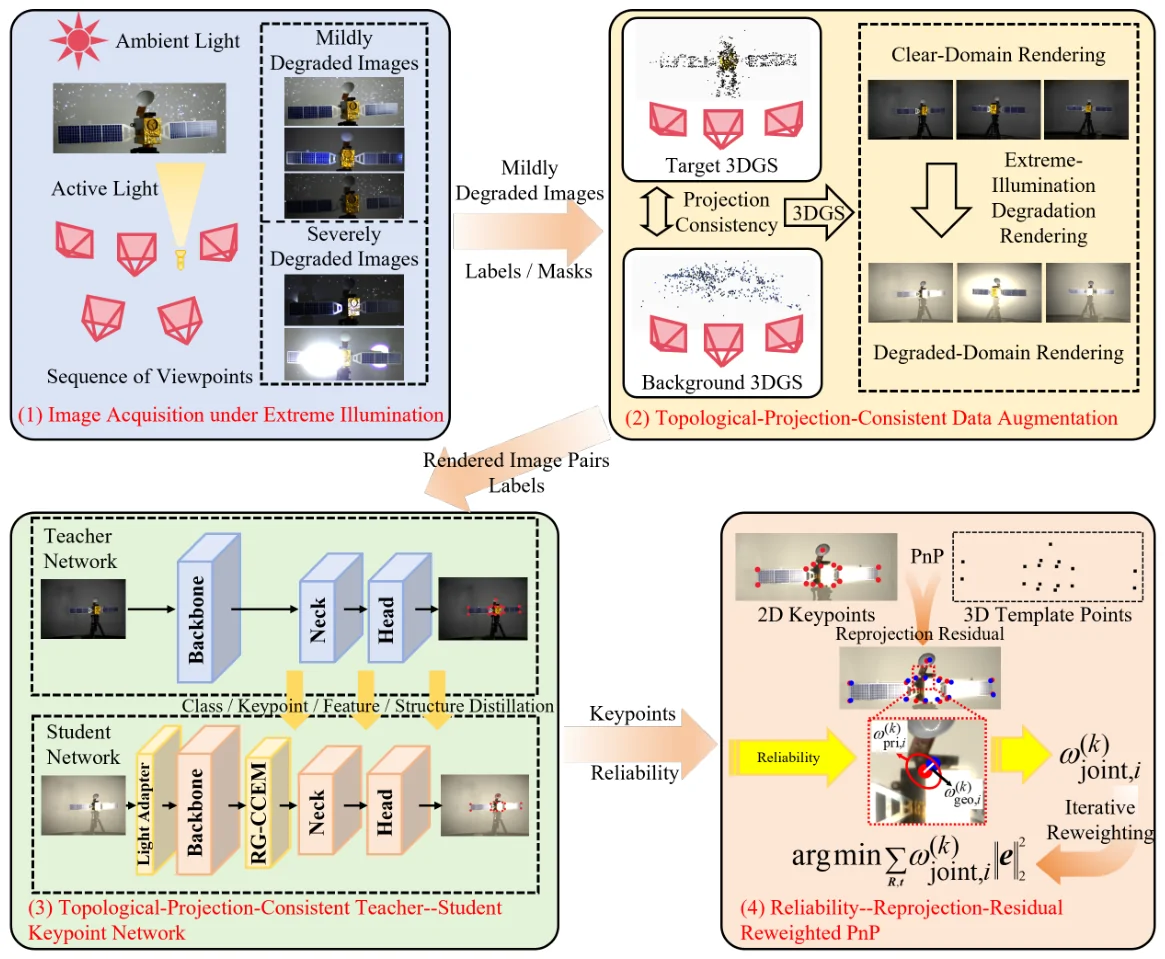
Framework of the proposed topological-projection-consistent augmentation, topological-projection-consistent distillation, and reliability–reprojection-residual reweighted pose-solving.

**Figure 2:**
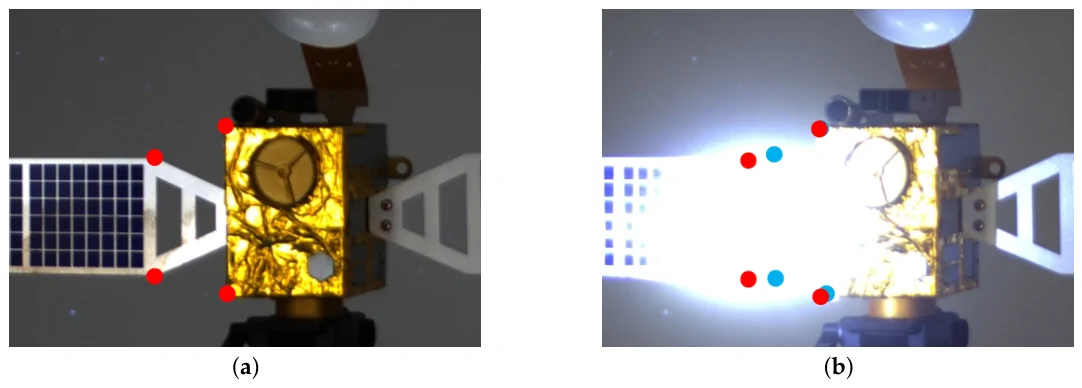
Examples of normal-illumination and extreme-illumination images. (**a**) Normal-illumination image. (**b**) Extreme-illumination image. Red points indicate ground-truth keypoints, and blue points indicate manually annotated keypoints.

**Figure 3:**
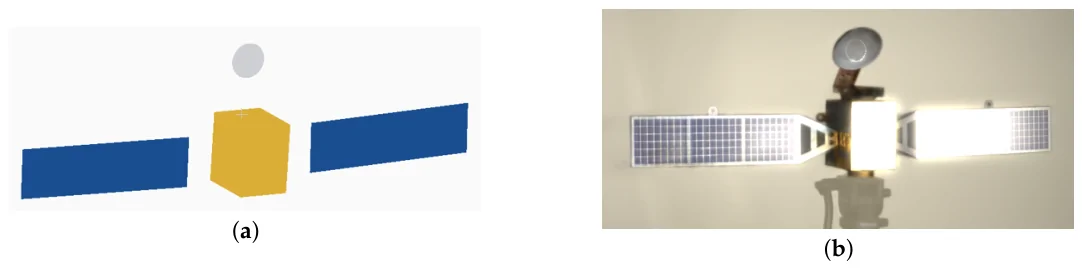
Proxy geometry and degraded rendering result. (**a**) Proxy geometry. (**b**) Extreme-illumination rendering result.

**Figure 4:**
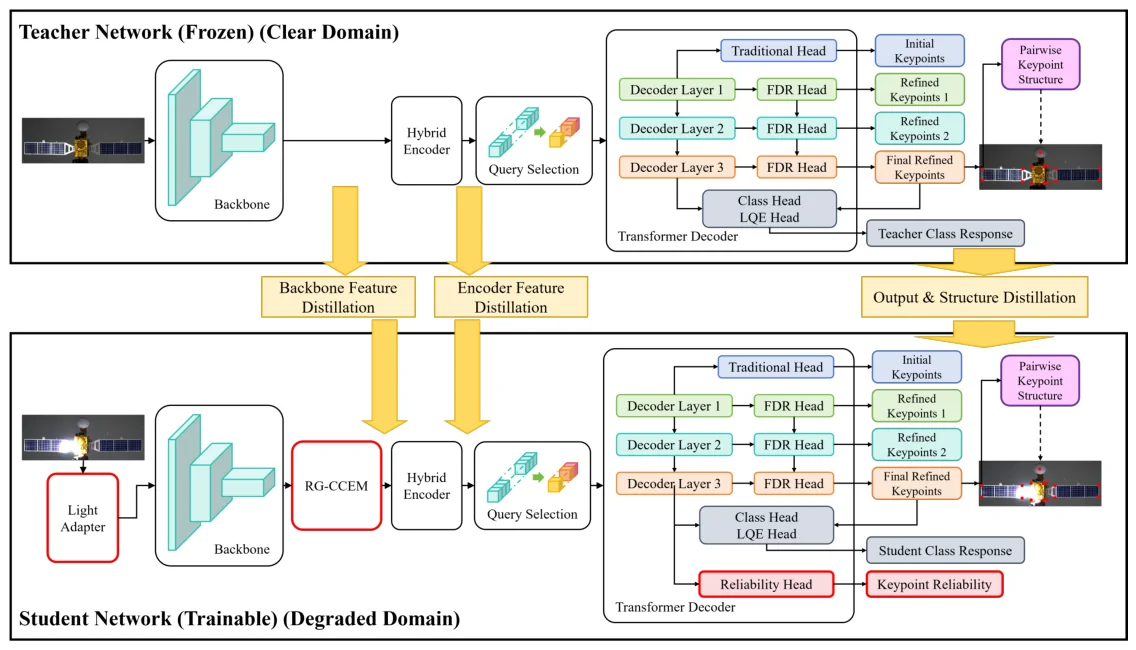
Teacher–student network framework for topological projection consistency.

**Figure 5:**
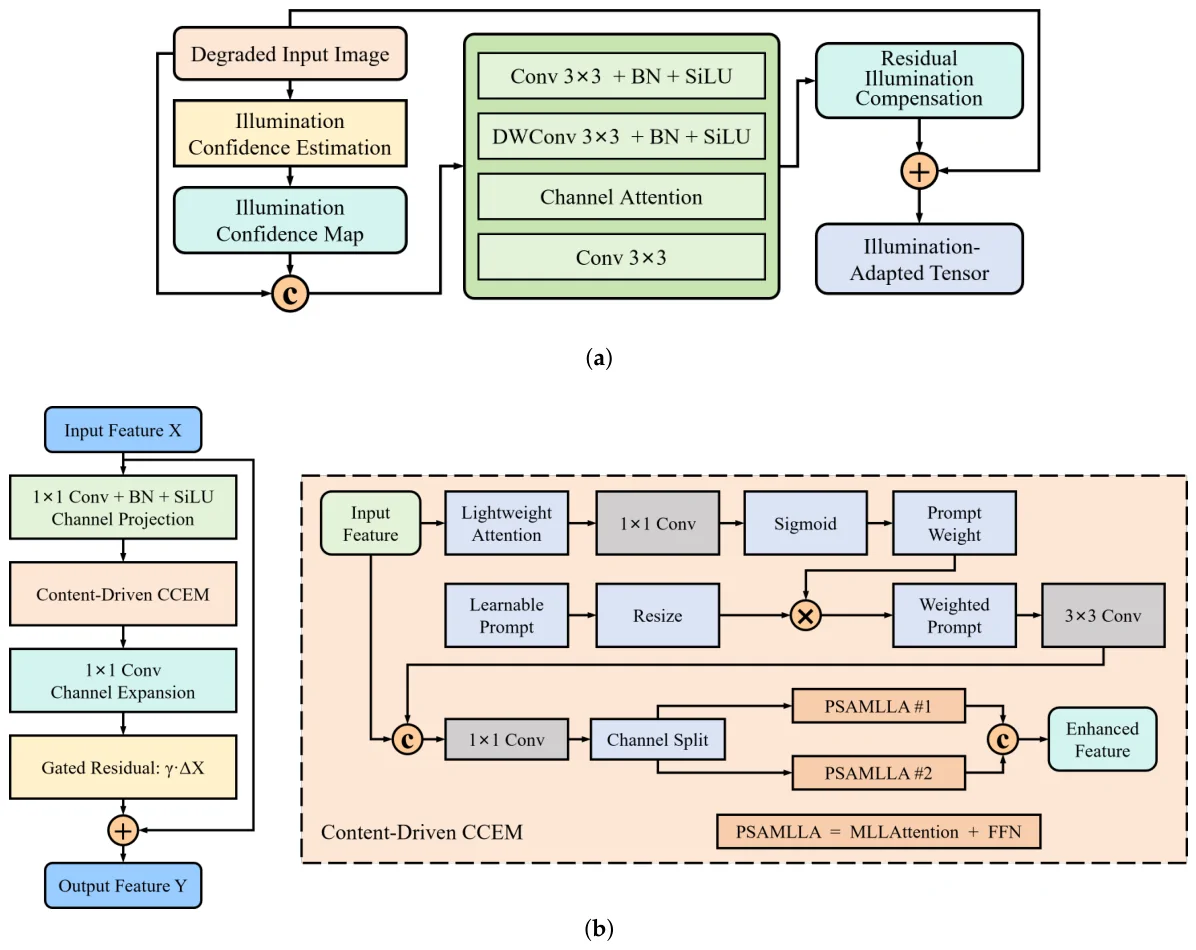
Feature compensation modules in the student network. (**a**) Light Adapter; (**b**) RG-CCEM.

**Figure 6:**
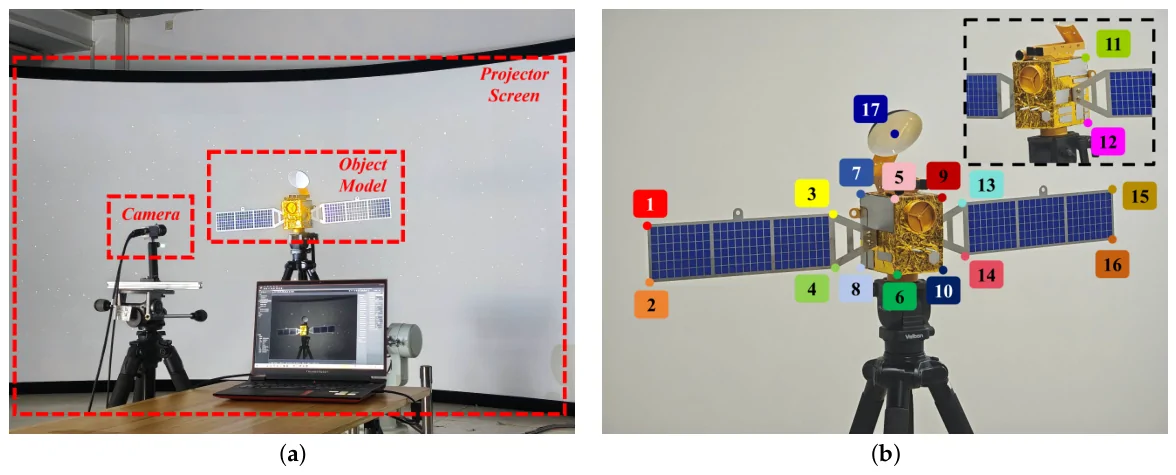
Experimental scene setup. (**a**) Experimental scene. (**b**) Target keypoints. (Different points are marked with different colors).

**Figure 7:**
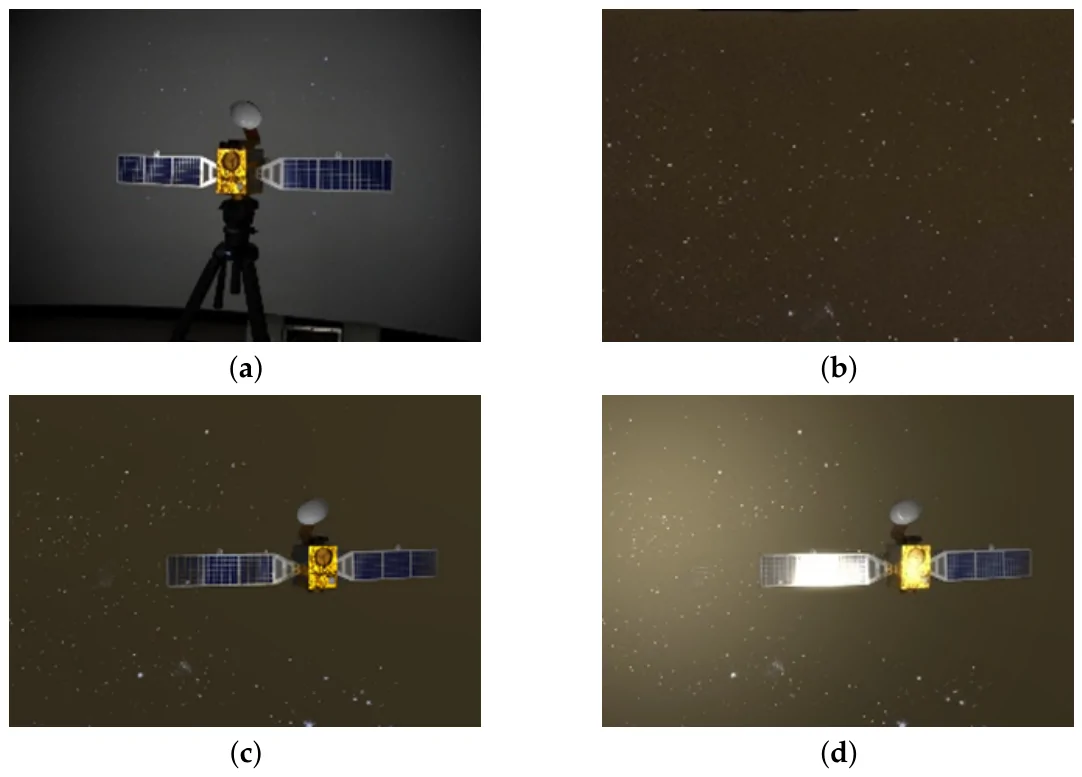
Examples of augmented data. (**a**) Mildly degraded image. (**b**) Background image. (**c**) Clear-domain rendering result. (**d**) Degraded-domain rendering result.

**Figure 8:**
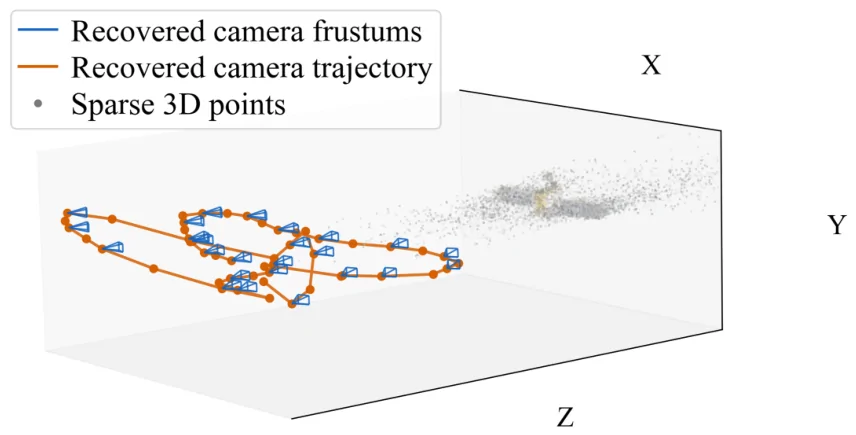
Sparse reconstruction result of augmented images. The red line indicates the novel-view trajectory, and the blue boxes indicate several view directions.

**Figure 9:**
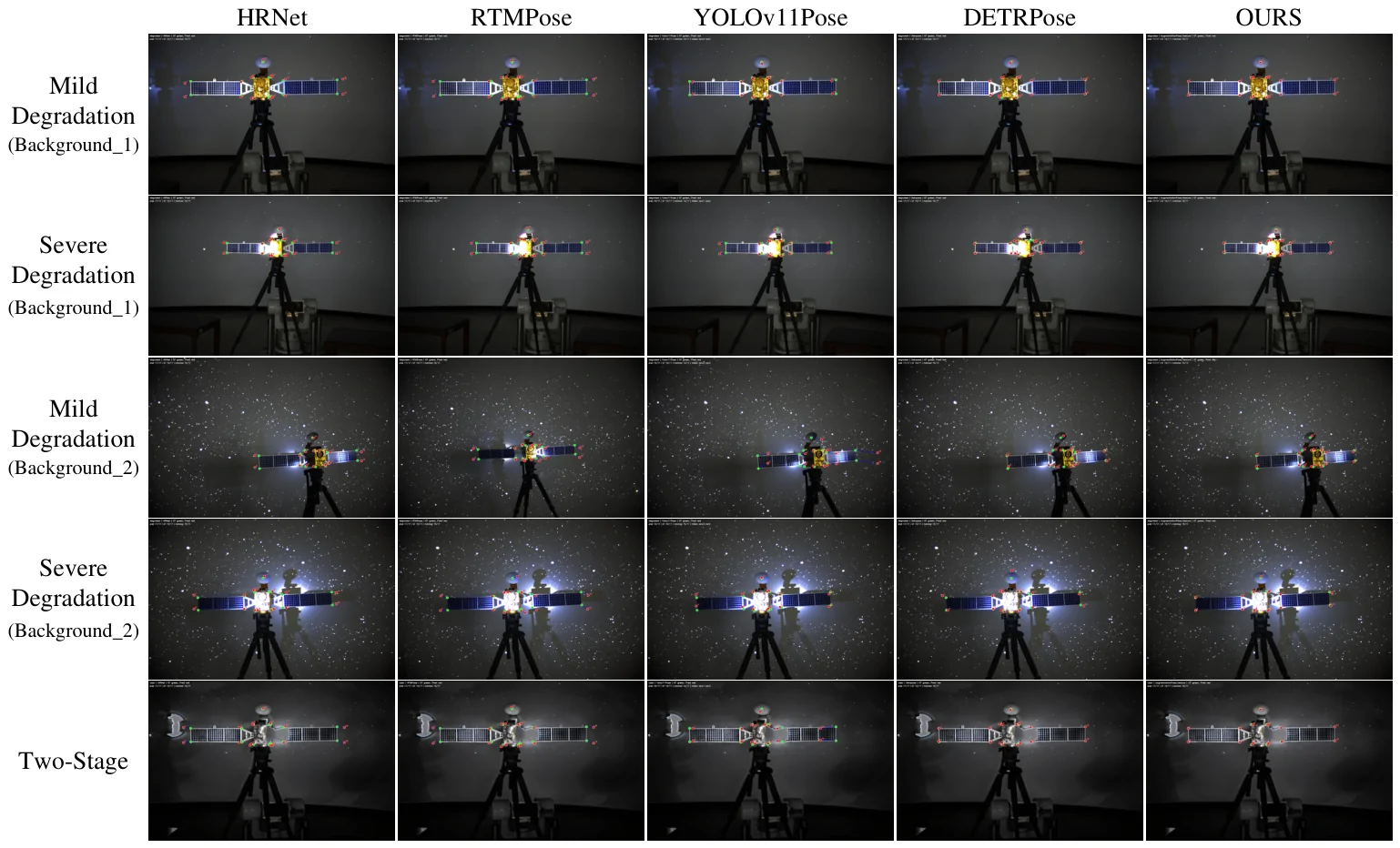
Keypoint extraction results. Green points indicate ground truth, and red points indicate detection results.

**Figure 10:**
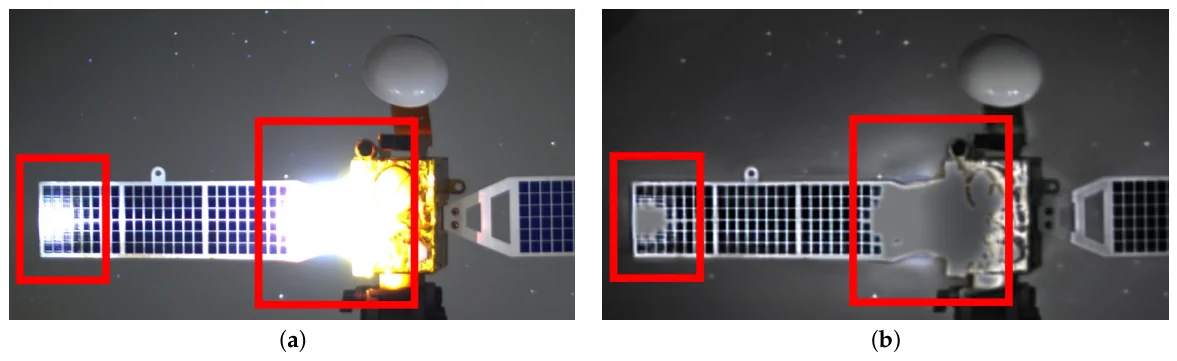
Extreme-illumination image and its restoration result. (**a**) Extreme-illumination image. (**b**) CSEC restoration result. The red boxes indicate the Extreme-Illumination areas in the corresponding images.

**Figure 11:**
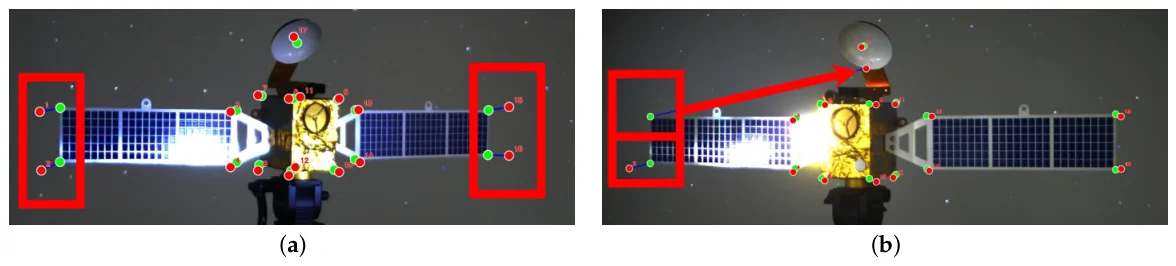
Abnormal extraction cases of RTMPose and YOLOv11Pose. (**a**) RTMPose. (**b**) YOLOv11Pose.

**Figure 12:**
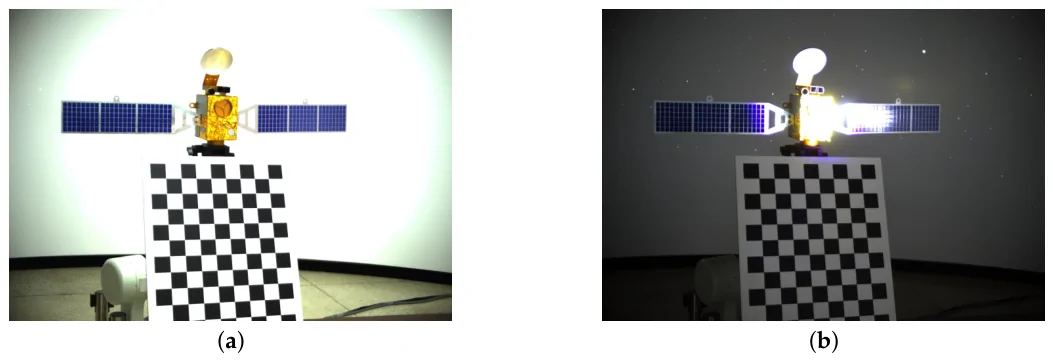
Ground-truth pose image and corresponding extreme-illumination image for pose testing. (**a**) Ground-truth pose image. (**b**) Extreme-illumination pose image.

**Figure 13:**
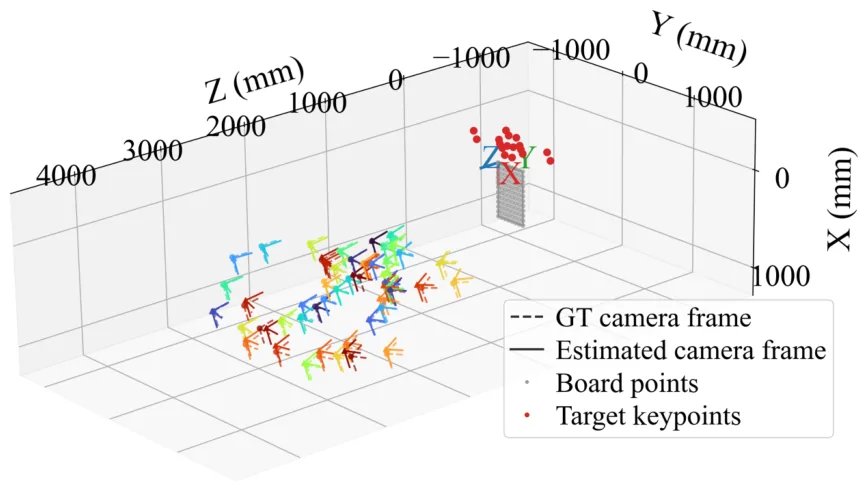
Visualization of pose measurement results. Dashed coordinate frames indicate ground-truth camera positions, and solid coordinate frames indicate measured positions. The coordinate axes of different colors represent the positional relationships of different measurement frames. The solid and dashed lines of the same color represent the positional relationship between the true value and the measured value at the same position.

**Figure 14:**
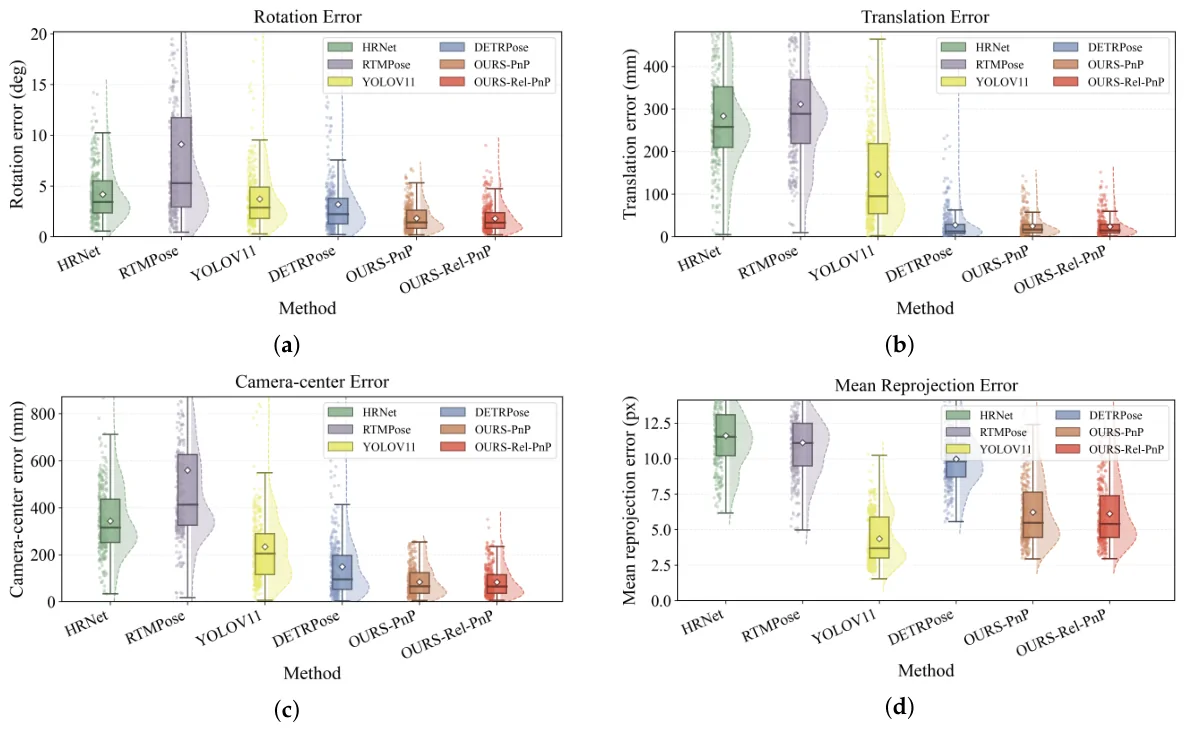
Pose error statistics. (**a**) Rotation error. (**b**) Translation error. (**c**) Camera-center error. (**d**) Reprojection error.

**Figure 15:**
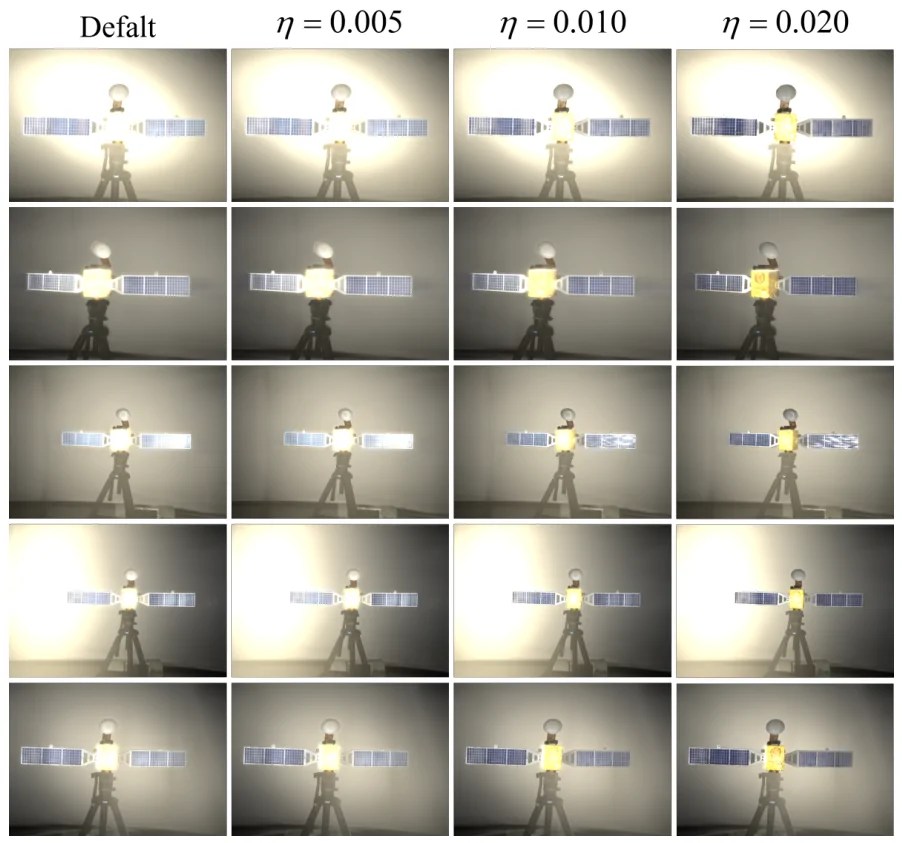
Comparison of rendering effects of proxy-geometry under different perturbations.

**Figure 16:**
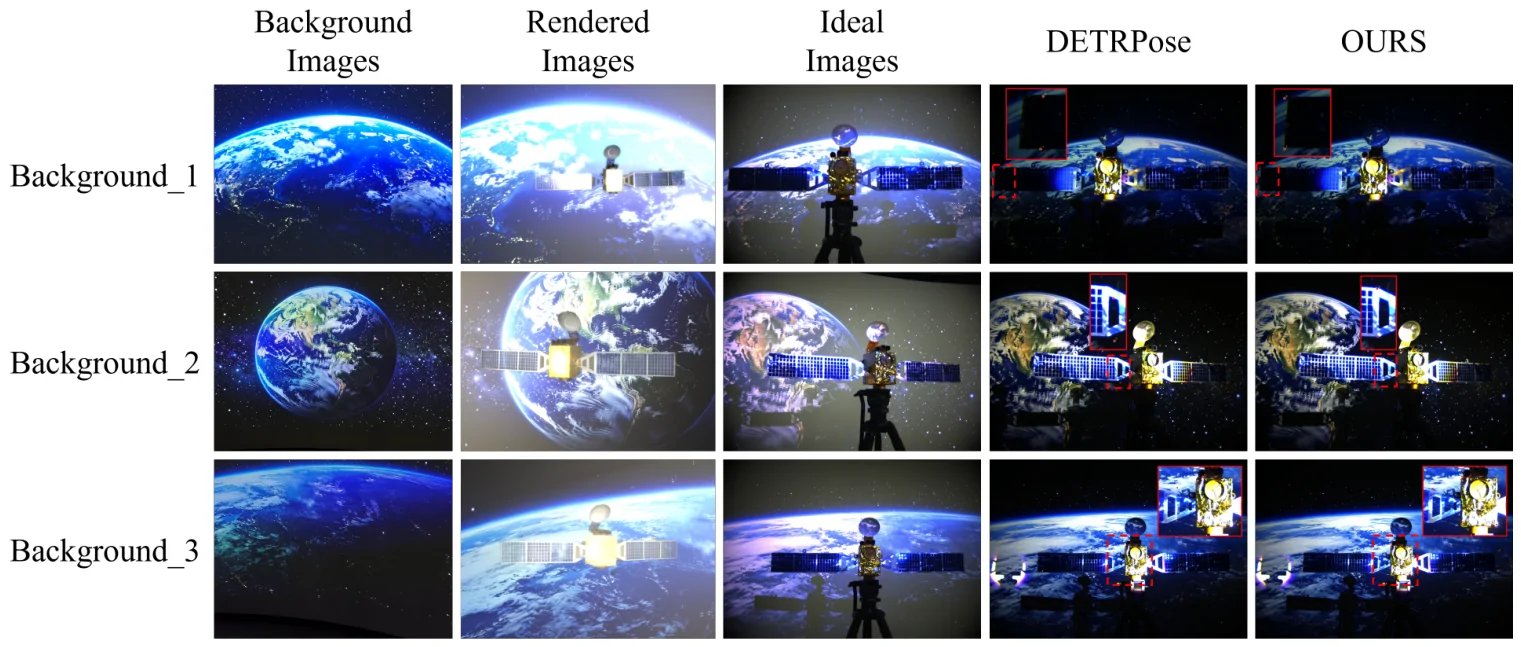
Visualization of the results of the cross-background experiment. The first column contains the background images. The second column contains the rendered images. The third column shows the acquired images with ideal lighting conditions. The fourth and fifth columns show the DETRPose and the proposed extraction results, respectively, where the local features are magnified. The red box shows a magnified view of local keypoint extraction.

**Table 1:** Comparison of keypoint detection results and outlier-separated pixel errors on all extreme-illumination images. (Bold and underlined values denote the best and second-best results, respectively).

Stage	Method	AP50 ↑	AP75 ↑	AP50:95 ↑	MPE_≤50_ (px) ↓	RMSE_≤50_ (px) ↓	Error >50 px ↓
One-stage	HRNet	0.796	0.277	0.352	24.31	27.28	1325 (29.8%)
One-stage	RTMPose	0.623	0.008	0.155	25.92	28.66	1980 (44.6%)
One-stage	YOLOv11Pose	0.771	0.283	0.359	23.19	26.37	1402 (31.6%)
One-stage	DETRPose	0.918	0.480	0.555	19.98	23.51	934 (21.0%)
Two-stage	HRNet	0.600	0.025	0.217	24.36	27.03	1915 (43.1%)
Two-stage	RTMPose	0.327	0.008	0.077	26.80	29.61	2334 (52.6%)
Two-stage	YOLOv11Pose	0.341	0.127	0.163	23.88	27.20	2124 (56.4%)
Two-stage	DETRPose	0.796	0.378	0.425	20.26	23.81	1275 (28.7%)
One-stage	Ours	**0.990**	**0.867**	**0.737**	**15.37**	**18.96**	**407 (9.2%)**

**Table 2:** Comparison of keypoint detection results on mildly degraded images. (Bold and underlined values denote the best and second-best results, respectively).

Stage	Method	AP50 ↑	AP75 ↑	AP50:95 ↑	MPE (px) ↓	RMSE (px) ↓
One-stage	HRNet	0.771	0.427	0.397	64.61	158.04
One-stage	RTMPose	0.670	0.036	0.207	52.78	64.73
One-stage	YOLOv11Pose	0.816	0.307	0.386	94.92	326.00
One-stage	DETRPose	0.963	0.524	0.582	38.48	70.02
One-stage	Ours	**1.000**	**0.899**	**0.779**	**20.91**	**33.27**

**Table 3:** Target-region photometric statistics for rendered degraded images, real degraded images, and real normal-illumination images. JS divergence and feature distance are computed with respect to the real degraded pool; lower values indicate closer distributional agreement.

Domain	*N*	Luma Mean	Saturated	Dark	Specular Proxy	Edge Mean	JS Luma ↓	Feature Dist. ↓
Real degraded	222	0.311	5.60%	4.30%	4.90%	0.0135	–	–
Rendered degraded	500	0.695	23.85%	0.00%	19.70%	0.0115	0.4791	1.140
Real normal	74	0.212	0.37%	8.19%	0.34%	0.0103	0.0841	1.271

**Table 4:** Comparison of pose measurement results. (Bold and underlined values denote the best and second-best results, respectively).

Method	Rotation Mean ↓ (deg)	Rotation RMSE ↓ (deg)	Translation Mean ↓ (mm)	Translation RMSE ↓ (mm)	Center Mean ↓ (mm)	Center RMSE ↓ (mm)	Reprojection RMSE ↓ (px)	Mean Inliers ↑
HRNet	4.19	4.93	283.45	311.18	343.92	370.03	13.30	14.65
RTMPose	9.11	13.22	311.23	343.98	558.81	688.68	13.07	14.04
YOLOv11Pose	3.72	4.74	146.63	215.88	234.03	300.72	**5.27**	14.74
DETRPose	3.19	4.58	27.59	48.94	149.48	219.26	11.74	14.57
Ours-DirectPnP	1.594	**2.05**	21.93	31.16	73.32	**94.97**	7.19	15.92
Ours-rel-PnP	**1.591**	2.06	**21.17**	**29.49**	**73.15**	95.84	7.53	**16.56**

**Table 5:** Single-realization proxy-geometry sensitivity results. The perturbation levels are controlled by the maximum displacement of non-keypoint vertices; the default proxy is the reference condition. AP is computed with the same stage-3 OKS sigma setting as the main keypoint-detection tables.

Condition	AP75 ↑	AP50:95 ↑	Outlier >50 px ↓	Rotation RMSE ↓ (deg)	Translation RMSE ↓ (mm)	Center RMSE ↓ (mm)
Default $P_{0}$	0.867	0.737	9.17%	2.05	31.16	94.97
η=0.005	0.864	0.728	9.75%	2.46	37.90	113.82
η=0.010	0.869	0.736	9.32%	2.74	36.14	128.23
η=0.020	0.817	0.704	11.60%	2.71	33.84	122.27

**Table 6:** Ablation results of the proposed modules and outlier-separated pixel errors. (Bold and underlined values denote the best and second-best results, respectively).

Variant	AP75 ↑	AP50:95 ↑	MPE_≤50_ (px) ↓	RMSE_≤50_ (px) ↓	Error >50 px ↓	Rotation RMSE ↓ (deg)	Translation RMSE ↓ (mm)	Center RMSE ↓ (mm)
Baseline	0.480	0.555	19.98	23.51	934 (21.0%)	4.58	48.94	219.26
TPCA only	0.847	**0.755**	**14.79**	**18.72**	**383 (8.6%)**	3.33	42.87	149.26
w/o RG-CCEM	0.835	0.720	16.20	19.96	430 (9.7%)	2.59	43.09	122.50
w/o SD	0.847	0.729	15.60	19.36	463 (10.4%)	2.34	38.66	108.73
Full	**0.867**	0.737	15.37	18.96	407 (9.2%)	**2.05**	**31.16**	**94.97**

**Table 7:** Compact diagnostic comparison between TPCA only and Full.

Evaluation Object	Metric	TPCA Only	Full	Full–TPCA Only
Keypoint test set	MPE_≤50_ (px)	14.79	15.37	+0.58
Keypoint test set	RMSE_≤50_ (px)	18.72	18.96	+0.24
Keypoint test set	Outlier ratio	8.6%	9.2%	+0.6 pp
Pose subset	Common-keypoint MPE (px)	10.13	8.90	−1.23
Pose subset	Common-keypoint RMSE (px)	15.84	14.05	−1.79
Pose images	Images with lower rotation error	123/319	196/319	+73
Pose images	Images with lower translation error	119/319	200/319	+81
Pose images	Images with lower center error	124/319	195/319	+71

**Table 8:** Backend-control comparison between TPCA only and Full under residual-only IRLS refinement. (Bold and underlined values denote the best and second-best results, respectively).

Variant	Backend	Rotation RMSE ↓ (deg)	Translation RMSE ↓ (mm)	Center RMSE ↓ (mm)
TPCA only	DirectPnP	3.33	42.87	149.26
TPCA only	Residual-only IRLS	3.29	40.77	146.65
Full	DirectPnP	2.05	31.16	94.97
Full	Residual-only IRLS	**1.98**	**27.91**	**90.76**

**Table 9:** Cross-background keypoint detection results on the aggregated test set from three additional backgrounds. (Bold values denote the best results).

Method	AP50 ↑	AP75 ↑	AP50:95 ↑	MPE_≤50_ (px) ↓	RMSE_≤50_ (px) ↓	Outlier >50 px ↓
DETRPose	**0.9540**	0.2357	0.4025	28.91	31.34	41.83%
Ours	0.9299	**0.4471**	**0.4746**	**21.53**	**24.94**	**30.37%**

**Table 10:** Hyperparameter sensitivity analysis and isolated $L_{3D}$ ablation. Frontend metrics use the complete 296-image keypoint test set, whereas pose RMSE is computed on the same 319-image subset for every row. The disabled $L_{3D}$ variant produces one additional catastrophic pose failure on the separately reported image. For backend-only parameters, the keypoint predictions are unchanged.

Hyperparameter	Setting	AP75 ↑	Outlier >50 px ↓	Rotation RMSE (deg) ↓	Translation RMSE (mm) ↓	Center RMSE (mm) ↓
All	default (main)	0.867	9.17%	2.04	29.38	95.54
$\lambda_{\mathrm{pair}}$	0.5× (0.04)	0.860	9.30%	2.56	38.37	125.69
$\lambda_{\mathrm{pair}}$	2× (0.16)	0.830	10.00%	2.63	36.48	131.88
$\lambda_{3D}$	disabled (0)	0.795	12.00%	3.01	35.40	149.76
$\lambda_{3D}$	0.5× (0.01)	0.864	8.74%	2.79	45.84	136.56
$\lambda_{3D}$	2× (0.04)	0.844	8.60%	3.51	71.16	173.77
$\lambda_{\mathrm{rel}}$	0.5× (0.175)	0.874	8.78%	2.90	41.73	144.09
$\lambda_{\mathrm{rel}}$	2× (0.70)	0.863	8.76%	2.71	40.97	139.52
$\tau_{r}$	0.5× (4 px)	0.867	9.17%	2.04	29.10	95.27
$\tau_{r}$	2× (16 px)	0.867	9.17%	2.05	30.10	96.12
$\omega_{\min}$	0.5× (0.10)	0.867	9.17%	2.03	29.36	94.82
$\omega_{\min}$	2× (0.40)	0.867	9.17%	2.07	29.43	96.78

**Table 11:** Topological projection consistency and paired statistical significance on the 320-image pose test set. The *p*-value corresponds to the paired two-sided *t*-test for ${\mathrm{TPC}}_{\mathrm{GT}}$ between each baseline and Ours. (Bold values denote the best results).

Method	${\mathrm{TPC}}_{\mathrm{GT}}$ (px) ↓	${\mathrm{TPC}}_{\mathrm{Proc}}$ (px) ↓	MPE_≤50_ (px) ↓	RMSE_≤50_ (px) ↓	Outlier >50 px ↓	*p*-Value vs. Ours
HRNet	69.71	25.58	27.67	29.95	40.28%	<$10^{-4}$
RTMPose	87.98	35.66	29.08	31.30	56.01%	<$10^{-4}$
YOLOv11Pose	586.81	548.31	21.07	23.20	29.89%	<$10^{-4}$
DETRPose	30.80	27.91	13.82	16.60	9.58%	<$10^{-4}$
Ours	**15.20**	**11.38**	**8.07**	**9.71**	**2.41%**	—

**Table 12:** rel-PnP stress analysis grouped by number of valid predicted keypoints. *N* denotes successful pose estimates over all images in the subset. (Bold values denote the best results).

Valid-Keypoint Bin	Backend	*N*	Rotation RMSE (deg) ↓	Translation RMSE (mm) ↓	Center RMSE (mm) ↓
$n_{\mathrm{valid}}\leq 14$	DirectPnP	28/29	2.35	33.45	97.65
$n_{\mathrm{valid}}\leq 14$	Residual-only IRLS	28/29	2.35	32.30	96.90
$n_{\mathrm{valid}}\leq 14$	rel-PnP	29/29	3.29	50.39	145.48
$15\leq n_{\mathrm{valid}}\leq 16$	DirectPnP	126/126	2.35	38.25	110.60
$15\leq n_{\mathrm{valid}}\leq 16$	Residual-only IRLS	126/126	2.25	33.21	103.72
$15\leq n_{\mathrm{valid}}\leq 16$	rel-PnP	126/126	**2.15**	**30.54**	**102.44**
$n_{\mathrm{valid}}=17$	DirectPnP	165/165	1.72	23.87	80.48
$n_{\mathrm{valid}}=17$	Residual-only IRLS	165/165	1.67	**22.06**	78.21
$n_{\mathrm{valid}}=17$	rel-PnP	165/165	**1.66**	22.97	**77.98**

**Table 13:** rel-PnP stress analysis grouped by initialization quality. Poor initialization corresponds to the worst 25% of images by initial RANSAC mean reprojection residual (≥7.136 px). (Bold values denote the best results).

Initialization Bin	Backend	*N*	Rotation RMSE (deg) ↓	Translation RMSE (mm) ↓	Center RMSE (mm) ↓
Poor init (worst 25%)	DirectPnP	80/80	2.81	48.49	130.04
Poor init (worst 25%)	Residual-only IRLS	80/80	2.63	40.91	**118.98**
Poor init (worst 25%)	rel-PnP	80/80	**2.61**	**38.32**	121.70
Well-init (remaining 75%)	DirectPnP	239/240	1.72	22.56	79.86
Well-init (remaining 75%)	Residual-only IRLS	239/240	**1.71**	**21.90**	**79.09**
Well-init (remaining 75%)	rel-PnP	240/240	1.83	25.88	85.50

**Table 14:** Measured model complexity, single-image inference speed, and training wall-clock time for the DETRPose family used in the proposed pipeline.

Method	Params (M)	MACs (G)	FLOPs (G)	Latency (ms)	Inference FPS (TITAN V)	Mean Epoch Time (s)	Training Time (h)
Teacher (ours)	4.07	4.61	9.26	29.28	34.15	42.16	6.50
Student (ours)	4.47	5.23	10.56	48.48	20.63	106.21	8.44
DETRPose baseline	4.07	4.61	9.26	29.28	34.15	78.31	6.55

## Data Availability

The data presented in this study are available from the corresponding author upon reasonable request.
